# Trimetallic Oxide Electrocatalyst for Enhanced Redox Activity in Zinc–Air Batteries Evaluated by In Situ Analysis

**DOI:** 10.1002/advs.202303525

**Published:** 2023-10-02

**Authors:** Ramasamy Santhosh Kumar, Pandian Mannu, Sampath Prabhakaran, Ta Thi Thuy Nga, Yangsoo Kim, Do Hwan Kim, Jeng‐Lung Chen, Chung‐Li Dong, Dong Jin Yoo

**Affiliations:** ^1^ Department of Energy Storage/Conversion Engineering of Graduate School (BK21 FOUR) Hydrogen and Fuel Cell Research Center Jeonbuk National University Jeonju Jeollabuk‐do 54896 Republic of Korea; ^2^ Research Center for X‐ray Science Department of Physics Tamkang University Tamsui 25137 Taiwan; ^3^ Department of Nano Convergence Engineering Jeonbuk National University Jeonju Jeonbuk 54896 Republic of Korea; ^4^ Korea Basic Science Institute Jeonju Center Jeonju‐si Jeollabuk‐do 54896 Republic of Korea; ^5^ Division of Science Education and Institute of Fusion Science Jeonbuk National University Jeonju Jeollabuk‐do 54896 Republic of Korea; ^6^ National Synchrotron Radiation Research Center Hsinchu 30076 Taiwan; ^7^ Department of Life Science Jeonbuk National University Jeonju‐si Jeollabuk‐do 54896 Republic of Korea

**Keywords:** alkaline Zinc‐air battery, in situ measurements, intrinsic activity, oxygen reduction/evolution reactions, trimetallic catalysts

## Abstract

Researchers are investigating innovative composite materials for renewable energy and energy storage systems. The major goals of this studies are i) to develop a low‐cost and stable trimetallic oxide catalyst and ii) to change the electrical environment of the active sites through site‐selective Mo substitution. The effect of Mo on NiCoMoO_4_ is elucidated using both in situ X‐ray absorption spectroscopy and X‐ray diffraction analysis. Also, density functional theory strategies show that NiCoMoO_4_ has extraordinary catalytic redox activity because of the high adsorption energy of the Mo atom on the active crystal plane. Further, it is demonstrated that hierarchical nanoflower structures of NiCoMoO_4_ on reduced graphene oxide can be employed as a powerful bifunctional electrocatalyst for oxygen reduction/evolution reactions in alkaline solutions, providing a small overpotential difference of 0.75 V. Also, Zn–air batteries based on the developed bifunctional electrocatalyst exhibit outstanding cycling stability and a high‐power density of 125.1 mW cm^−2^. This work encourages the use of Zn–air batteries in practical applications and provides an interesting concept for designing a bifunctional electrocatalyst.

## Graphical abstract

Both experimental and theoretical methods are used to examine how well spinel to tri‐metallic catalysts via zinc–air batteries perform in processes involving oxygen reduction and oxygen evolution reactions.

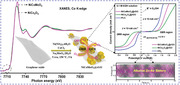

## 1 Introduction

Developments in energy storage and conversion are necessary due to the increasing global energy demand and environmental issues. Fuel cells,^[^ ^1^, ^2^ ^]^ electrolyzers of water,^[^ ^3^, ^4^, ^5^ ^]^ metal‐air batteries,^[^ ^6^, ^7^ ^]^ and energy conversion technologies are only a few examples. Rechargeable zinc‐air batteries have attracted a much attention due to their high energy density (1086 Wh kg^−1^), the natural abundance of zinc, low cost, and acceptable protection (alkaline electrolyte).^[^ ^8^, ^9^ ^]^ However, modern Zn–air batteries have some issues such as poor round‐trip effectiveness, unstable cycling, significant deterioration of graphite catalysts, and low density of the charging‐discharging current. The fundamental cause of these issues are the absence of a long‐lasting, high‐performance catalyst that can accelerate both oxygen reduction and evolution reactions (ORR and OER, respectively).^[^ ^10^ ^]^ To produce high‐performance rechargeable Zn–air batteries, it is crucial to construct a greater ORR/OER bifunctional catalyst with better activity and durability.^[^ ^11^ ^]^

Spinel oxides (AB_2_O_4_) were recently demonstrated to have a variety of uses in electrode materials and energy storage.^[^ ^12^ ^]^ Among the most interesting combined bimetal oxide is NiCo_2_O_4_, in which the Ni and Co cations occupy octahedral and tetrahedral/octahedral sites, respectively. The redox combination Co^2+/3+^/Ni^2+/3+^ gives NiCo_2_O_4_, which shows an electrocatalytic activity twice as high as that of NiO and Co_3_O_4_.^[^ ^13^, ^14^ ^]^ The greater electrocatalytic activity of binary oxide over that of mono‐metal oxide is due to the increased electrical conductivity of the material.^[^ ^15^, ^16^ ^]^ However, NiCo_2_O_4_ alone has a weak catalytic activity for applications in energy storage because of its low pore volume and cycling instability.^[^ ^17^ ^]^ Hence, creating multimetallic oxides is a potential method to increase the activity of electrocatalysts due to the interactions between various metal components can efficiently change the 3d electronic structure, resulting in improved oxygenated intermediate adsorption‐free energies that boost electrocatalytic activity.^[^ ^18^, ^19^ ^]^ For example, newly developed trimetallic electrocatalysts like Ir‐NiCo_2_O_4_,^[^ ^20^ ^]^ Fe─Co─Ni‐MOF,^[^ ^21^ ^]^ Spinel NiCo_2−x_Fe_x_O_4_,^[^ ^18^ ^]^ MnNiCo_2_O_4_,^[^ ^22^ ^]^ MgCr_2_O_4_,^[^ ^23^ ^]^ Ni─Fe─MoN NTs,^[^ ^24^ ^]^ and FeCoMoS@NrGO^[^ ^25^ ^]^ were studied, and they mostly promoted OER or ORR on many active sites of different metals. Therefore, It is crucial to modify trimetallic oxide using a variety of techniques, such as adding activated carbons, conducting polymers, nanomaterials, or doping heteroatoms to improve the bimetal oxides' electrochemical performance.^[^ ^26^ ^]^ Recently, doping of transition metals has been offered as a viable alternative to achieve better electrochemical performance because they can modify the electronic structure and enhance electrical conductivity.^[^ ^27^ ^]^ Thus, it is desirable to synthesize well‐defined hierarchical trimetallic oxides with optimal intrinsic activity and a large number of exposed active sites.

Here, we describe a simple hydrothermal approach for preparing a trimetallic catalyst on reduced graphene oxide (NiCoMoO_4_@rGO). Since Ni^2+^ and Co^3+^ ionic radii are comparable to those of Mo^6+^ (a high‐valence non‐3d transition metal ion^[^ ^28^ ^]^), they were added to a NiCo‐layered double hydroxide (NiCo‐LDH). The produced catalyst retained hierarchical nanoflower structures, has a high degree of crystallinity, and is chemically stable, all of which can enhance mass transfer and increase accessibility of active sites. The NiCoMoO_4_@rGO electrocatalyst performed well as OER and ORR electrocatalysts, requiring only a 332 mV overpotential for OER at 10 mA cm^−2^ and an *E*_1/2_ = 0.81 V for ORR. We conducted density functional theory (DFT) calculations for the predicted Mo atoms in the active site. The rechargeable Zn–air battery assembly bifunctional OER/ORR electrocatalyst generated an impressive power density of 125.1 mW cm^−2^ and an impressive specific capacity of 976 mAh g^−1^. The prepared catalyst were thoroughly characterized using operando X‐ray absorption and in situ X‐ray diffraction investigations (for ORR and Zn–air batteries). This study demonstrates innovation in the design, synthesis, and synergistic effects of non‐noble metal catalysts. Finally, exceptional energy storage capacity and bifunctional catalytic abilities for OER/ORR can open up new paths for the development of energy conversion and storage technologies.

## 2 Results and Discussion

### 2.1 Physicochemical Characterization

**Figure 1:**
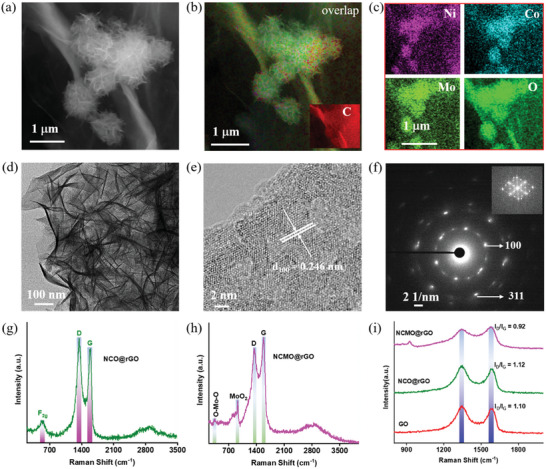
a) SEM image, b,c) SEM‐EDS elemental mapping for corresponding Ni, Co, Mo, and O atoms in the NCMO HNFs catalyst. d) TEM, e) HR‐TEM, and f) SAED pattern (inset image shows the corresponding FFT image) of NCMO HNFs catalyst. g–i) Raman analysis of NCO@rGO and NCMO@rGO catalysts.

**Scheme 1:**
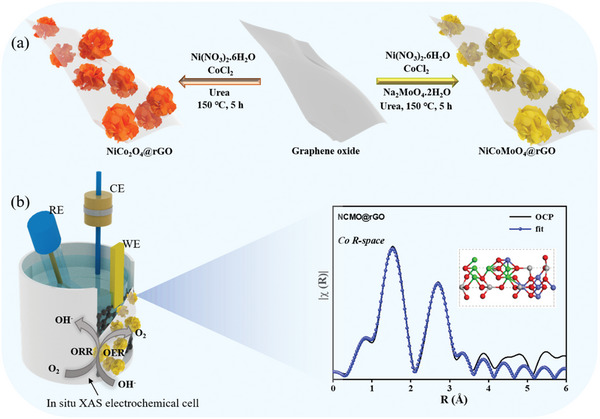
a) Schematic illustration of the synthesis of NiCo_2_O_4_ and NiCoMoO_4_ on supported graphene oxide, b) typical experimental setup for an in situ XAS analysis.

As shown in **Figure** **1**a, the NiCo_2_O_4_ (NCO) and NiCoMoO_4_ (NCMO) on rGO were prepared using a hydrothermal technique at 150 °C for 5 h, employing nickel nitrate, cobalt chloride, and sodium molybdate as metal precursors. The development of NCO and NCMO on rGO was controlled in this instance using ammonium fluoride as a structural tuning agent and urea promotes the change of GO to rGO. Therefore, the surface structure of NCO and NCMO on rGO was revealed by SEM and TEM analysis. Figure S1a (Supporting Information) demonstrates the fact that FE‐SEM image of GO shows an even surface of sheets of GO with numerous wrinkles on sizes of a few micrometers. Also, the functional groups in GO produce strong contacts with the metal precursors and improve the conductivity. First, we conform NCO bimetal catalyst synthesis; as a result, the TEM image in Figure S1b (Supporting Information) shows the hierarchical nanoflower structure (HNFs) of NCO produced on the rGO sheet (NCO@rGO). Further, the formation of HNF structures crystal planes is clearly displayed in Figure S1c (Supporting Information) of the TEM image, it shows a (Ni/Co) atom arrangement that is suitable with the (422) crystal plane. TEM‐EDS elemental maps and EDS spectrum clearly demonstrate the formation of NCO on rGO as shown in Figure S1d,e (Supporting Information). Additionally, we added Mo atoms in the NCO catalyst using the same synthesis method, which catalyst had an identical form to that of NCO; as a result, the NCMO catalyst was successfully formed, as shown in Figure 1a. Constructed on the HNF structure of SEM image and the corresponding SEM‐EDS elemental mapping presented in Figure 1b,c. Subsequently reach out that the constituent elements Ni, Co, Mo, and O are uniformly distributed across the HNF structures in the NCMO catalyst formation (**Scheme** **1**a).

Thereafter, the TEM image clearly shows the hierarchical structure of the NCMO on supported rGO nanosheet, as in Figure 1d, Figure S2a (Supporting Information). Also, Figure S2b (Supporting Information) illustrates the interparticle connection between the adjacent HNF structure of NCMO on rGO. This interconnected nanostructure can boost the electron transport for the complete HNFs while strengthening the tensile stability during electrocatalysis. In combination, the atomic arrangement of the atoms was very similar to the NCMO (100) plane crystal lattice in Figure 1e. Therefore, the selected area electron diffraction (SAED) patterns (Figure 1f) support this by displaying a ring with a bright spot that reveals the polycrystalline nature of HNF‐structured NCMO, which further indicates the (100) and (311) planes. The HNF‐structured NCMO catalyst was crystalline in nature as supported by Fast Fourier Transform (FFT) data (inset image of Figure 1f).

The trimetallic HNF structure of NCMO@rGO obtained by high‐angle annular dark‐field scanning transmission electron microscopy (HAADF‐STEM) allows for clear visualization of the metal atoms (Ni, Co, and Mo) (Figure S3a,b, Supporting Information). Moreover, TEM‐EDS mapping images support the creation of HNF structures and the dispersion of Ni, Co, Mo, and O atoms on an atomic level. The Mo atoms were homogeneously distributed in the trimetallic catalyst on rGO nanosheets (Figure S3c–f, Supporting Information), and EDS spectra clearly showed the formation of NCMO based on the presence of Ni, Co, Mo, O, and C (Figure S3g, Supporting Information). Further, we examine the weight ratios of Ni, Co, and Mo in NCO@rGO and NCMO@rGO catalysts were calculated in ICP‐OES, indicating loadings of Ni, Co, and Mo shown in Figure S3h,i (Supporting Information). Moreover, a type IV isotherm indicative of HNF materials can be seen in the N_2_ equilibrium adsorption of NCO@rGO and NCMO@rGO (Figure S4a,b, Supporting Information). In HNF structures of NCMO on the rGO nanosheet, Mo provides a stable basis.^[^ ^28^, ^29^ ^]^ Particle bonding was supported by the hierarchical structure and shape of HNFs to provide a larger surface area on the catalyst layer. As a result, the activated HNF‐structured NCMO had a higher BET surface area than NCO@rGO (64.1 m^2^ g^−1^), indicating a greater number of accessible active sites. The increased pore size distribution in the BJH desorption process (13.6 and 13 nm) is expected to lead to improved electrochemical activity (Figure S4c,d, Supporting Information).^[^ ^30^ ^]^ The in situ and ex situ XAS studies provides the evidence for the catalytic activity by identifying more active regions.

Raman analysis was used to examine the structural and defect properties of the composites GO, NCO@rGO, and NCMO@rGO. Figure 1g presents Raman spectra of the as‐prepared NCO@rGO composite and one obvious peak at 534 cm^−1^ (F_2g_) was observed in the Raman spectra.^[^ ^31^ ^]^ After the addition of the Mo atom, displayed stretching modes of doubly coordinated oxygen are attributed to the NCMO@rGO Raman peak at 345 cm^−1^ (O─Mo─O),^[^ ^32^ ^]^ as shown in Figure 1h. Also, the polarization characteristics of the 923 cm^−1^ band precisely match the vibrational characteristics anticipated for a dioxo unit, and they are attributed to Mo═O (MoO_2_) stretching mode.^[^ ^33^ ^]^ These vibrational modes confirm the conversion of NCO to NCMO composites. Additionally, Figure 1i depicts the Raman spectra of NCO@rGO and NCMO@rGO catalysts, revealing distinct peaks of the D band at 1342 cm^−1^ (attributed to defects in graphitic carbon) and the G band at 1581 cm^−1^ (attributed to the sp^2^ nature of carbon atoms in the catalyst). The *I*_D_/*I*_G_ values of the NCO@rGO catalyst were ≈1.12, which was somewhat lower than that of the NCMO@rGO catalyst (*I*_D_/*I*_G_ = 0.92), suggesting a favorable association between NCO@rGO, NCMO@rGO, GO, and successful formation of catalysts.^[^ ^26b^ ^]^

According to Figure S5a (Supporting Information), X‐ray diffraction analysis was used to determine the phase and crystal structure of NCO@rGO and NCMO@rGO catalysts. Based on the XRD patterns, it is clear that Mo has an important effect on the crystallinity of the catalyst. The NCO@rGO catalyst shows crystalline (JCPDS: 073–1704) with the characteristic diffraction peaks at 31.0⁰ (311), 33.4⁰ (222), 47.1⁰ (422), and 59.8⁰ (442).^[^ ^34^ ^]^ After the addition of Mo atom in the NCMO@rGO catalyst, strong crystalline peaks appeared at 36.1⁰ (100) on the key crystal plane, respectively (JCPDS: 050–0739).^[^ ^35^ ^]^ These results demonstrate the successful conversion of spinel to a trimetallic catalyst.

### 2.2 Surface Oxidation and Local Structure

**Figure 2:**
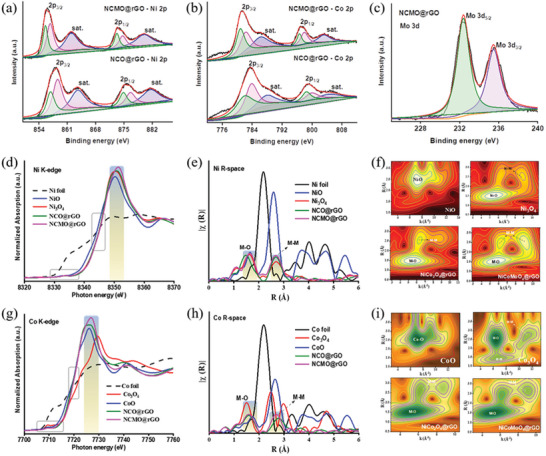
XPS spectra for a) Ni 2p spectra, b) Co 2p spectra of NCO@rGO and NCMO@rGO, and c) Mo 3d spectra of NCMO@rGO. d,e) XANES and EXAFS spectra for Ni foil, NiO, Ni_3_O_4_, NCO@rGO, and NCMO@rGO, (f) Corresponding wavelet transforms for the k3‐weighted Ni K‐edge. g,h) XANES and EXAFS spectra for Co foil, CoO, Co_3_O_4_, NCO@rGO, and NCMO@rGO, i) Corresponding wavelet transforms for the k3‐weighted Co K‐edge.

XPS spectra were obtained to determine the catalyst valence states and chemical structure of the catalysts. As seen in Figure S5b (Supporting Information), the XPS survey spectra for spinel NCO@rGO and NCMO@rGO demonstrate the inclusion of Ni 2p, Co 2p, Mo 3d, C1s, and O 2p signals. The NCO@rGO composite Ni 2p spectrum showed Ni 2p_3/2_ and Ni 2p_1/2_ at 858 and 875 eV, respectively, consistent with the literature and showed the presence of Ni^2+^/Ni^3+^ in this composite (**Figure** **2**a).^[^ ^18^, ^30^ ^]^ Moreover, after the addition of Mo atoms in the spinel catalysts, the XPS peak binding energies slightly changed for Ni 2p_3/2_ (855 eV) and Ni 2p_1/2_ (872 eV). The XPS spectra of Co 2p show peaks at 783 and 798 eV in Figure 2b, which correspond to Co 2p_3/2_ and Co 2p_1/2_ in spinel catalyst, respectively; these can be compared with the NCMO@rGO composite showing XPS peaks at 781 (2p_3/2_) and 797 eV (2p_1/2_).^[^ ^4^, ^36^, ^37^, ^38^ ^]^ As seen in Figure 2c, the Mo 3d spectra of NCMO@rGO indicate the presence of Mo 3d_5/2_ (232 eV) and Mo 3d_3/2_ (235 eV) species, indicating a lower oxidation state for NCMO@rGO. This shows the successful formation of NCMO@rGO composite.^[^ ^36^, ^39^ ^]^ Additionally, Figure S5c (Supporting Information) depicts the O 1s spectrum, which clearly shows the M─O bonds in before and after doping of Mo atom in the spinel catalyst. Figure S5d (Supporting Information) depicts the C 1s spectrum, which shows the M─C bonds before and after doping of Mo atoms in the spinel catalyst. Peak tables of NCO@rGO and NCMO@rGO composites also display the changes in binding energy and atomic ratio (Figure S25a,c, Supporting Information).

### 2.3 Ex Situ XAS Analysis

The electrocatalytic activity is expected to be strongly related to the local and electronic structures of Ni, Co, and Mo in the prepared catalysts. Therefore, the detailed local atomic and electronic structures of the active catalysts were examined using X‐ray absorption near edge structures (XANES) and extended X‐ray absorption fine structures (EXAFS), which are sensitive to the local electronic structure.^[^ ^40^, ^41^, ^42^ ^]^ Figure 2d displays the Ni K‐edge XANES spectra of NCO@rGO and NCMO@rGO along with the standard reference Ni foil, NiO and Ni_3_O_4_. The main absorption peak, a relatively small pre‐edge peak of NCO and NCMO Ni K‐edge indicate that both catalyst structures were mostly centrosymmetric.^[^ ^43^, ^44^ ^]^ It seems that the main absorption peak is slightly shifted to higher energy for both NCO@rGO and NCMO@rGO compared to the standard reference NiO (+2 oxidation state) suggesting the existence of more Ni^2+^. However, the absorption edge and the main absorption peak are located at a higher energy for NCMO@rGO compared to NCO@rGO, indicating a higher valent (Ni^3+^) state. In fact, the pre‐and‐rising edge also shifted to a higher energy than the NiO and NCO@rGO, indicating the increased oxidation state in the NCMO@rGO.

In the case of Co K‐edge, the energy position, and pre‐edge peak intensity are associated with the local symmetry and oxidation state of Co.^[^ ^45^ ^]^ The pre‐edge peak (≈7709 eV) intensity is found to be low (inset in Figure 2g) and the intensity of the main absorption peak (≈7726 eV) is high for NCMO@rGO indicating the presence of more octahedral coordinated Co^3+^, and the local structure around Co becomes more centrosymmetric.^[^ ^45^ ^]^ Similar to the Ni K‐edge XANES spectra, the absorption edge NCMO@rGO is slightly shifted to higher energy, and the main absorption peak is also located at higher energy compared to the NCO@rGO suggesting Co in the NCMO@rGO has a more positive oxidation state than NCO@rGO. It seems that adding Mo to NCO@rGO eventually changes the valence state of the Ni/Co ions as evidenced by the Co and Ni K‐edge spectra. Moreover, Co K‐edge spectra of NCMO@rGO shifted to higher energy indicating the partial transformation of Co^2+^ to Co^3+^ which could be the reason for the enhanced ORR activity of NCMO@rGO in the present work. Additionally, the observed Co and Ni K‐edge XANES spectra of NCMO@rGO reveal the spinel structure, octahedral and tetrahedral Ni/Co ions are occupied by the trivalent and divalent metal ions, respectively.^[^ ^34^, ^46^ ^]^ Therefore, the excellent electrochemical performance of NCMO@rGO could be due to the existence of spinel structures with the majority of Co^3+^ and Ni^3+^ at octahedral sites.^[^ ^47^ ^]^

Further, R‐space FT‐EXAFS spectra of NCO@rGO show two dominant peak features at ≈1.48 and ≈2.68 Å that are assigned to the Ni─O and Ni─Ni coordination, respectively (Figure 2e). Interestingly, the decreased Ni─O bond length in NCMO@rGO (≈1.64 Å) compared to NiCo_2_O_4_@rGO (≈1.48 Å) indicates the structure transformation from NCO@rGO to trimetallic compound (NCMO@rGO). This decreased M─O bond length after the Mo substitution which was caused by structural contraction (as seen in Figure S17, Supporting Information).^[^ ^48^ ^]^ The existence of higher oxidation state of NCMO@rGO could be attributed to the higher coordination number of Ni─O and also to the introduction of Mo─O, which subtracted electrons from Ni centers through Ni─O─Co/Mo bond. Furthermore, Figure 2h displays the Fourier‐transformed R‐space spectra of the Co K‐edge for NCO@rGO and Mo‐doped NCMO@rGO catalysts along with standard reference Co foil, CoO, and Co_3_O_4_. In the case of NCO@rGO, two main peaks located at ≈1.57 and ≈2.77 Å originate from Co─O and Co_oh_─Co_oh_ bonds respectively.^[^ ^49^, ^50^, ^51^ ^]^ Co─O and Co─Co shell coordination indicate that Co^3+^ ions are octahedrally coordinated. The NCMO@rGO sample also shows two primary peaks at ≈1.57 and ≈ 2.69 Å corresponding to Co─O and Co_oh_─Co_oh_ bonds. Notably, the Co_oh_─Co_oh_ bond length decreased for NCMO@rGO which could be due to the oxidation from Co^2+^ to Co^3+^, which led to a decrease in the radii of Co ions. Also, this contraction in the Co─Co bond may be attributed to the existence of rich cation vacancies.^[^ ^49^, ^50^, ^51^ ^]^ The observed results suggest the presence of more oxidation Co^3+^ in the NCMO@rGO which seems to agree with Co K‐edge results.

To further explore the atomic structure of the catalyst, the corresponding k^3^χ data of the EXAFS oscillations of Ni k‐space and Co k‐space were performed. Figure S6a,b (Supporting Information) shows the different EXAFS oscillations for both NCO@rGO and NCMO@rGO which indicates the different local atomic environments around Ni and Co in all the samples. Moreover, the different EXAFS oscillations for both NCO@rGO and NCMO@rGO indicate the different coordination geometry around Ni/Co sites. Also, this obvious difference indicates that the introduction of Mo significantly influenced the local coordination environment of Ni/Co atom. To better understand the characteristics of nearby metal atoms, wavelet transform (WT)‐EXAFS was carried out (Figure 2f,i and Figure S6c,d, Supporting Information). The presence of M─O and M─M bonds can be seen in NCO@rGO and NCMO@rGO, while Co─O and Ni─O bonds are visible in the WT‐EXAFS spectrum of the Co and Ni edges respectively. These results suggest the occurrence of Ni, Co, and Mo species of trimetallic oxide catalysts.

### 2.4 In Situ XAS Analysis in ORR Reaction

**Figure 3:**
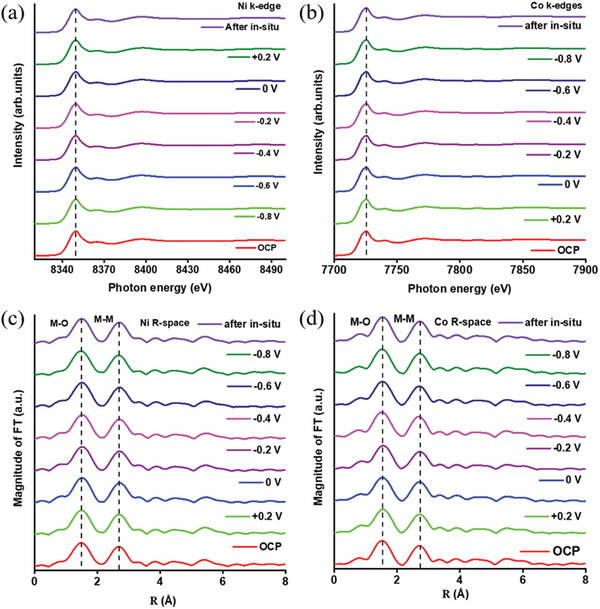
In situ study for a,b) XANES spectra of Ni and Co K‐edge, (c, d) EXAFS spectra of Ni and Co K‐edge for NCMO@rGO electrocatalyst at OCP and ‐0.8 V applied potential.

**Figure** **3**a–d displays the in situ XANES and EXAFS spectra of NCMO@rGO at the Ni and Co K‐edges, which can be used to determine the active sites of Ni/Co/Mo atomic pairs. From the Ni K‐edge (Figure 3a), the absorption peak position exhibits a slight shift with an applied voltage increase (see Table S2, Supporting Information for OCP and after ORR of Ni─O and Ni─Ni bond), this clearly demonstrates an increase in the Co ion valency (Co^2+^ → Co^3+^) in ORR environments. A small contribution from the surface‐active layer that is partially replicated as in Co K‐edge XANES spectra captured in mass transmission mode is responsible for this slight rise in the valence state of Co (Figure 3b). Also, the Co ion in NCMO@rGO undergoes slightly faster oxidation and shows considerable change in the absorption edge position at −0.80 V compared to that of Ni in NCMO@rGO at an applied voltage at over −0.60 V, signifying an increase in the Co valence.^[^ ^52^ ^]^ The absorption edge shows a small energy shift at the center of the spectrum with an increase in the voltage to −0.80 V (from −0.60 V to −0.80 V), further confirming a slight increase in the Co valence. Moreover, Ni K‐edge spectra does not show a significant change in the absorption edge position (Figure S7c,d, Supporting Information), further confirming Co is a more active center for ORR. The overlap of Ni K‐edge ranges or the absorption edge positions at the different operating voltages indicate the inactive nature of Ni ions during the ORR, as presented in the inset of Figure 3a.^[^ ^53^ ^]^

Figure S7a,b (Supporting Information) contrasts the pre‐edge regions of the Ni and Co K‐edges, which pre‐edge features in both of these spectra display 1s to 3d electronic transitions. The difference in peak intensity and the absorption edge position often offer structural information that contains the local atomic symmetry and oxidation states of Co.^[^ ^42^, ^45^, ^54^ ^]^ The pre‐edge feature at 7709 eV in the Co K‐edge is similar to CoO, which suggests the octahedral coordination of Co. The weak pre‐edge features of both Co and Ni K‐edges XANES spectra suggests Ni/Co atoms occupy the octahedral sites, indicating that both oxides exhibit a mostly spinel structure. Additionally, the pre‐edge peak intensity at Co K‐edge (at 7709.5 eV) is slightly increased after ORR that corresponds to the 1s → 3d transition allowed for Co^2+^ ions occupying the Td sites (Figure S7b, Supporting Information).^[^ ^45^, ^55^ ^]^ Likewise, the XANES spectra at the Ni K‐edge for the NiCoMoO_4_@rGO before and after the ORR condition displays a decrease in the pre‐edge peak (at 8332.5 eV) (Figure S7a, Supporting Information). This shows that Co or Ni ions in the Td site decreased while Co or Ni ions in the Oh site increased. However, the Ni K‐edge XANES spectra of NiCoMoO_4_@rGO displayed an insignificant change in the absorption edge positions after the ORR, evidence that the Ni persisted as Ni^2+^ during the ORR. The similar EXAFS results for the Ni K edge (i) and Co K edge (j) indicate Co and Ni are coordinated in the octahedral structure (Figure 3c,d). The bond distance of the first (Co─O) and second shell (Co─Co/Ni/M) is very similar to that of Ni─O and Ni─Ni/Co/M bond distances, further confirming that Ni─Ni/Co/M is octahedrally coordinated. The primary peak at ≈1.50 Å in Figure 3d corresponds to Co─O bonds that consist of an octahedral Co^3+^−O (O_h_) shell and a tetrahedral Co^2+^−O (T_d_) shell. The second peak (at ≈2.70 Å) is related to Co─Co bonds for two octahedral Co ions, while the third peak (≈3.30 Å) is associated to Co─Co bonds for two tetrahedral Co (Co_Td_‐M_Td_) or tetrahedral and octahedral Co. (Co_Td_‐M_Oh_).

Generally, the coordination number can be estimated by multiplying the amplitude reduction factor (S_0_^2^) with N. The fitting results of Co K‐edge EXAFS analysis evidence that the coordination number (CN) of Co─O (5.02) was reduced at applied potential at −0.80 V compared to CN of Co─O (5.32) at open circuit potential (OCP) or at +0.20 V (CN of Co─O is 5.27) as shown in Figure S8 and Table S1 (Supporting Information), this change in the CN of Co─O at higher potential could be due to the oxygen or metal vacancies. It seems both the oxygen vacancies and metal vacancies coexisted as the coordination number is found to differ with an applied voltage for Co─O first shell and similar in Co─Co/Ni second shell. On the other hand, the fitting results of Ni K‐edge EXAFS analysis show an insignificant change or very similar CN for Ni─O and Ni─Ni bonds at different applied potentials (as shown in Figure S9 and Table S2, Supporting Information) suggesting that Ni^2+^ state remains same during the ORR activity. Even though, Co sites are believed to be the active sites among these elements. Mo sites are expected to be more active sites in the ORR activity (Mo XANES data not presented here) since the measured results indicate only a slight change in the CN of Co─O more often than Ni─O. Also, each CoO_6_ is loosely compacted with each other from the viewpoint of the Co site resulting in more structural disorder around the Co site. In other words, cobalt tends to change the local structural rearrangement and sustain the structure in the existence of oxygen or metal vacancies. According to the above in situ XANES results, metal or oxygen vacancies may extensively present in MO_6_ octahedra layers, giving rise to a relative structure disorder and more unbonded species within the layers, which creates more active sites in NCMO@rGO. Additionally, an electrochemical cell used in in situ XAS that uses laser X‐ray detection is schematically seen in Scheme 1b and Figure S10a–c (Supporting Information).

### 2.5 Ex Situ Electrochemical Oxygen Reduction Reaction (ORR)

**Figure 4:**
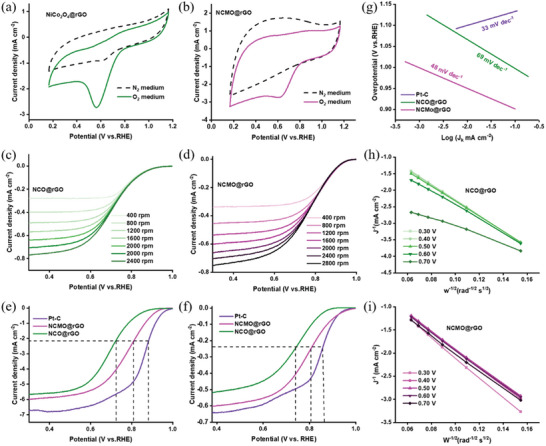
ORR performance of as‐obtained catalysts: a,b) ORR CV curves of the NCO@rGO and NCMO@rGO electrocatalysts, c,d) ORR LSV curves of the NCO@rGO and NCMO@rGO catalysts with different rotation speeds of RDE (400–2800 rpm), e,f) LSV curves of Pt─C, NCO@rGO, and NCMO@rGO before and after 5000 cycles of CV in 0.1 m O_2_ saturated KOH solution, g) Tafel plots Pt─C, NCO@rGO, and NCMO@rGO electrocatalysts, and h,i) corresponding *K*–*L* plots of NCO@rGO and NCMO@rGO electrocatalysts with different rotation speeds of RDE (400–2800 rpm).

The bifunctional oxygen catalytic performance of the electrocatalysts NCMO@rGO, NCO@rGO, Pt─C, and IrO_2_ was investigated, and the results showed good ORR/OER and Zn–air battery performance. We carefully analyzed the ORR reaction using both ex situ and in situ XAS studies to identify the active sites in NCMO@rGO electrocatalysts . Now, Pt wire, Ag/AgCl electrode, and NCMO@rGO were used as the counter, reference, and working electrodes, respectively. Cyclic voltammetry (CV) in 0.1 M  KOH saturated with N_2_ and O_2_ was used to examine the electrocatalytic activity of the catalysts created for the ORR. The catalyst reduction peak was absent in the N_2_ saturated solution, as shown in **Figure** **4**a,b, and Figure S11a (Supporting Information). On the other hand, once the electrolyte was saturated with oxygen, distinct cathodic reduction peaks emerged, whose intensity and position are indicative of the electrocatalytic activity of the ORR. With the exception of Pt─C (≈0.85 V), NCMO@rGO displayed a larger positive peak potential at ≈0.81 V versus the other catalyst (NCO@rGO; 0.77 V), emphasizing its superior ORR performance. Before, linear sweep voltammograms (LSVs) of various catalysts were recorded using a rotating disk electrode (RDE) at various speeds (400–2800 rpm) inside a 0.1 m O_2_ saturated KOH solution. Unless otherwise noted, the blank response that was recorded under N_2_ was used to correct the current density (Figure 4c,d and Figure S11b, Supporting Information). The ORR activity of NCO@rGO shown in Figure 4e and Figure S11c (Supporting Information) was significantly lower than that of the other materials (onset potential, *E*_onset_ = 0.86 V, and half‐wave potential, *E*_1/2_ = 0.78 V). This may be due to the stable state (Ni^3+^) that is present in the active sites and the severe aggregation of NCO@rGO during electrochemical testing.

However, the *E*_onset_ and *E*_1/2_ of NCO@rGO were greatly enhanced by the high‐conductivity GO, which also successfully prevented the aggregation of NCO@rGO. Additionally, NCMO@rGO outperformed NCO@rGO in terms of *E*_onset_ (0.89 V) and *E*_1/2_ (0.81 V). The majority of this is attributed to the trimetallic oxide catalysts having an abundance of active sites, which is realistic given the previously published articles listed in Table S6 (Supporting Information). Hence, NCMO@rGO showed superior values to the other synthetic materials, results that are nearly identical to that of commercial Pt─C (0.94 and 0.84 V). Furthermore, the trimetallic NCMO@rGO electrocatalyst limiting current (JL) was higher (4.3 mA cm^−2^) than that of the NCO@rGO electrocatalysts (3.41 mA cm^−2^) and is suitable for the benchmark Pt─C catalyst (4.6 mA cm^−2^) with its obviously faster reaction kinetics. Subsequently, long‐term stability is a key indicator of ORR characteristics, this catalyst was tested through 5000 constant CV cycles at a scan rate of 100 mV. When compared to commercial Pt─C, the catalytic activity of NCMO@rGO and NCO@rGO was marginally reduced, as demonstrated in Figure S12a–d (Supporting Information) (inset image: Figure S12d, Supporting Information). Figure 4f clearly displays the negative *E*_1/2_ shift of 14, 20 mV, which is lower than that of the commercial Pt─C catalyst (37 mV).

The Tafel slope was calculated for a manufactured material to evaluate the rate kinetics and define the reaction mechanism path of the electrochemical process. As shown in Figure 4g, the Tafel slope of NCMO@rGO (48 mV dec^−1^) was much lower compared to NCO@rGO (69 mV dec^−1^) and compared to that of Pt─C (33 mV dec^−1^), indicating that the kinetics at NCMO@rGO are fast. Moreover, LSV tests with different rotation speeds were carried out (along with the corresponding calculations) to explore the electron transport mechanism through the ORR. Figure 4h,i and Figure S11d (Supporting Information) show how current density rose with increasing speed of rotation and the Koutecky–Levich (*K*–*L*) connection was found for an NCMO@rGO altered RDE in the potential range of 0.3–0.7 V. The excellent linear relationships and nearly equal slopes of all the *K*–*L* plots, which were equivalent to the value of commercial Pt─C, indicate that the ORR can be modeled using a first‐order kinetics approach. The electron transport number for ORR at NCMO@rGO was 3.62 using the *K*–*L* equation, supporting a 4e^−^ ORR mechanism at the catalyst. Also, the non‐Faradaic zone CVs at various sweep speeds suggest that prepared electrocatalyst characteristics may be to blame for the large ECSA value, which is related to the C_dl_. Figure S13a–c (Supporting Information) demonstrates that NCMO@rGO had a significantly higher C_dl_ than NCO@rGO (2.9 mF cm^−2^), indicating that it has the greatest ECSA among the materials examined and, as a result, the most active sites, which helped provide the best ORR performance. Lastly, Electrochemical impedance spectroscopy (EIS) revealed a relatively fast energy transfer rate among NCMO@rGO and electrolyte species at the interface, as well as a low charge transfer resistance (*R*_ct_) in NCMO@rGO among the catalysts developed (Figure S13d, Supporting Information). These observations corresponded to the high stability and rapid kinetics of the NCMO@rGO electrocatalyst (inset image: Figure S13d, Supporting Information).

### 2.6 Electrochemical Oxygen Evolution Reaction (OER)

**Figure 5:**
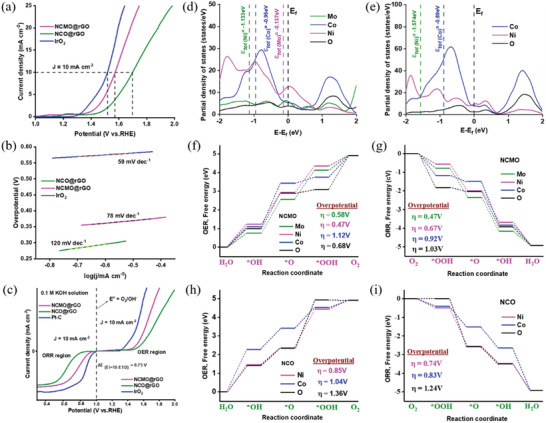
a) OER curves of polarization at 1 mV s^−1^ scan rate, b) Tafel slope, c) potential difference of NCMO@rGO, NCO@rGO, IrO_2_, and Pt─C. d,e) Spin‐polarized PDOS of the corresponding catalyst, Fermi level is set to zero, (f‐g) OER and ORR Gibbs free energy diagrams of NCMO, and (h‐i) NCO with its adsorption intermediates (*OH, *O, and *OOH).

After the ORR reactions, we measured the OER activity in a 0.1 M  KOH solution in a typical three‐electrode cell setup to explore the electrocatalytic activity of the trimetallic NCMO@rGO toward ORR/OER processes. **Figure** **5**a demonstrates that NCMO@rGO had the highest OER activity among these materials, with an *E*_onset_ of ≈1.47 V, making it more negative than the catalysts NCO@rGO (1.51 V) and IrO_2_ (1.43 V). A major additional parameter for determining the catalyst OER activity is the voltage needed to pass 10 mA cm^−2^ (*E*_j_ = 10). The NCMO@rGO electrocatalyst (1.52 V) was more effective for OER than those of NCO@rGO, as demonstrated in Figure S14a (Supporting Information), which displayed materials with sufficient OER activity in recently reported publications (Table S6, Supporting Information). Hereafter, the lower Tafel value of NCMO@rGO (78 mV dec^−1^) compared to that of NCO@rGO (120 mV dec^−1^) and the slightly lower Tafel value of IrO_2_ (59 mV dec^−1^) further demonstrated their faster reaction kinetics toward OER (Figure 5b).

Besides, the properties of the NCMO@rGO electrocatalyst may be the cause of the high ECSA, which is related to the C_dl_ determined from the CV at different sweep speeds in the non‐Faradaic zone. Figure S14b–e (Supporting Information) shows that NCMO@rGO (2.7 mF cm^−2^) had a substantially higher C_dl_ than NCO@rGO (1.6 mF cm^−2^), indicating that it had the largest ECSA and, consequently, the most active sites, which helped it achieve the highest OER performance. Also, EIS spectroscopy revealed a relatively fast charge transfer between NCMO@rGO and electrolyte species at the interface as well as a high *R*_ct_ value in NCMO@rGO among the catalysts generated, which demonstrates that the NCMO@rGO catalyst has outstanding OER electron‐transport kinetics and strong conductivity. Figure S14f (Supporting Information) illustrates the rapid kinetics and stability of the NCMO@rGO electrocatalyst displayed. In addition, TOF (turnover frequency) values of 0.0148 and 0.0159 s^−1^ were calculated for NCO@rGO and NCMO@rGO, respectively, using the ICP‐OES data. Hence, electrocatalysts with enhanced TOF values showed more reaction species and quicker electron/ion transport of NCMO@rGO electrocatalyst.^[^ ^5^ ^]^ The catalyst OER endurance was also tested for cyclic stability (Figure S15a–c, Supporting Information), and there was some change in the current density of NCMO@rGO (which is more oxidized compared to the initial to final cycles), showing that NCMO@rGO offers good cyclic durability. This is because the catalyst structure was carefully considered. In contrast, NCO@rGO and IrO_2_ are likely to be impacted by dissolution during the OER process. This result confirms the superior mass transport properties and electrode stability of NCMO@rGO catalyst, while Figure S15d (Supporting Information) shows after cyclic stability (at 500 mV scan rate) LSV curves that reveal a significant difference in the stability.

Finally, the Δ*E* value (Δ*E* = *E*_j = 10(OER)_−*E*_1/2(ORR_)) was utilized to compare the electrocatalysts ORR/OER dual‐functional behavior to that of the NCMO@rGO, NCO@rGO, and Pt─C electrocatalysts (Figure 5c). The bifunctional oxygen electrocatalyst efficiency and ΔE are often inversely related. The aggregate ORR/OER polarization curves of the various catalysts (Table S6, Supporting Information) show that NCMO@rGO had the lowest Δ*E* value at 0.752 V, with the exception of Pt─C + IrO_2_. Lastly, a DFT study of the material was also carried out to determine how the catalytically active metal affects the bifunctional properties.

### 2.7 Density Functional Theory Simulation

We used DFT calculations to examine how the incorporation of Mo atom alters the NCO catalytic performance. To determine the most stable NCMO, four alternative configurations were considered, as shown in Figure S16 (Supporting Information), and the values of the different configurations are given in Table S3 (Supporting Information). As shown in Table S3 (Supporting Information), the Mo doping leads to more negative formation energy (*E*_F_), indicating it is more stable than NCO. Configuration 2 shows the lowest *E*_F_ among the four configurations. For the optimized bulk structure, the catalyst was cleaved at the 311 plane (the major plane) according to XRD data shown in inset image Figure S17a,b (Supporting Information). We analyzed the E_F_ for both the catalysts, which showed a more negative E_F_ for the optimal catalyst NCMO, the values are presented in Table S4 (Supporting Information). The electronic structure is one of the key indicators of electron transfer properties, and this information can be used to determine the physical origin of the catalytic activity of the materials. The catalytic activity is predicted by the *d*‐band center theory.^[^ ^56^, ^57^, ^58^ ^]^According to this theory, the strength of the metal‐hydrogen (H) interaction is used to determine where the *d*‐band center lies relative to the Fermi level (E_f_). In general, antibonding states have a higher energy than *d‐*states, so the energy level (ε*_d_*) of the model would be a good description of the metal interactions.^[^ ^59^ ^]^ When more antibonding states are filled, the downward shift results in a weaker bond. Figure 5d,e demonstrates the PDOS results for NCMO and NCO. The figure shows that the *d*‐band center of Mo is located at −0.137 eV from *E*_f_, while Ni and Co are located at −1.133 and −0.95 eV, respectively, a little farther from E_f_. These values can be compared to the *d*‐band centers related to Ni and Co in NCO located at −1.574 and −0.89 eV, respectively. As a result of optimal position of the *d*‐band center at Mo, NCMO may produce higher conductivity and high adsorption capacity during ORR, enabling intermediates to be more readily adsorbed.

According to Norskov and co‐workers, each ORR elementary step involves a free energy change (△*G*) based on the computational hydrogen electrode (CHE) model.^[^ ^60^, ^61^ ^]^ Figures S18 and S19 (Supporting Information) depict the optimized adsorption structures of the catalyst with adsorption intermediates *OOH, *O, and *OH. The Gibbs free energy diagram is shown in Figure 5g,i, and the values are presented in Table S5 (Supporting Information). The ORR overpotential obtained for each active site of the catalyst of NCMO shows a theoretical overpotential of 0.47 V at an active site Mo, whereas other active sites of Ni, Co, and O were 0.66, 0.91, and 1.03 V, respectively. The active site of NCO has a higher overpotential than NiCoMoO_4_, suggesting the improvement of ORR activity by the incorporation of Mo.

For comparison, we calculated OER mechanism at the active sites located on the surface of NCMO and NCO (Figure 5f,h). Among the theoretical OER overpotential obtained for each active site of the catalyst of NCMO and NCO, the optimal catalyst NCMO delivers a superior overpotential of 0.47 V at an active site “Ni” whereas other active sites of Mo, Co, and O deliver 0.58, 1.12, and 0.68 V, respectively. The theoretical ORR overpotential of 0.47 V was obtained preferentially at the active site of “Mo” at the catalyst NCMO, implying that the ORR occurs at a different active site when compared to OER and recently published articles.^[^ ^25^ ^]^ The theoretical results clarify that the introduction of “Mo” into NCO plays a key role in reducing the overpotential and optimizes the energy barriers in the reaction steps to enhance the catalytic activity in case of ORR.

### 2.8 Zn‐Air Battery Assembly

**Figure 6:**
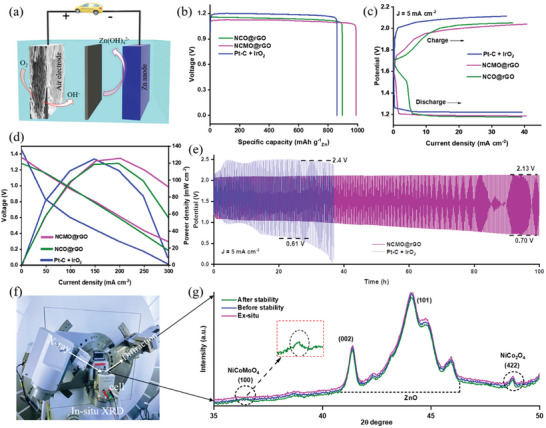
Performances of Zn–air batteries using an air cathode: a) The fabrication of a zinc‐air battery is shown in the schematic diagram. b) Specific capacity of a zinc‐air battery with air cathodes of Pt─C + IrO_2_, NCMO@rGO, and NCO@rGO operating at 10 mA cm^−2^. c) NCMO@rGO, NCO@rGO, and Pt─C + IrO_2_ air cathode current density curves at 5 mA cm^−2^. d) Charts representing the power density and discharge polarization of a zinc‐air battery with an air cathode of NCMO@rGO, NCO@rGO, and Pt─C + IrO_2_. e) Zn–air batteries with an NCMO@rGO and Pt─C + IrO_2_‐based air cathode having a long‐term galvanostatic charge–discharge with 5 min charged and 5 min discharged at a current density of 5 mA cm^−2^. f,g) The setup for in situ XRD analysis and the occurrence of the X‐ray diffraction peak took place in the Zn–air battery.

A rechargeable Zn–air battery was built using NCO@rGO, NCMO@rGO, and Pt─C + IrO_2_ as the air electrode due to its outstanding ORR and OER performances. In a Zn–air battery, a Zn plate serves as the anode, and the NCMO@rGO electrocatalyst is employed as the air cathode, which is present in the 6 M  KOH containing zinc acetate (0.2 M ) electrolyte as shown in **Figure** **6**a.^[^ ^62^, ^63^ ^]^ Comparative testing also was performed using the commercial Pt─C + IrO_2_ catalyst with an identical setup. The open‐circuit voltage (OCV‐1.41 V) of the liquid Zn–air battery, as shown in Figure S20a,b (Supporting Information), was slightly greater than that of the Pt─C + IrO_2_ and was very different than that of NCO@rGO (1.388 V), proving that the trimetallic catalysts electrocatalytic activity was increased by the presence of Mo. Furthermore, a light‐emitting diode (LED) was illuminated by connecting two liquid Zn–air batteries together, as shown in Figure S20c (Supporting Information).

When subjected to identical current densities, the NCMO@rGO‐based Zn–air battery exhibited a higher voltage platform than Pt─C + IrO_2_. The difference in discharge voltage is more apparent under increasing current densities. Additionally, the battery based on NCMO@rGO has a specific capacity and correlated energy density (standardized to the mass of consumed Zn) that are assessed to be 976 mAh g^−1^ (Figure 6b), which are significantly higher than those of the NCO@rGO (850.2 mAh g^−1^) and Pt─C + IrO_2_ (796.2 mAh g^−1^). Because of this, a range of technological devices may employ the developed Zn–air batteries. Hence, charge‐discharge polarization curves (Figure 6c) show a reduced voltage difference compared to the NCO@rGO and Pt─C + IrO_2_ catalyst because NCMO@rGO electrocatalysts indicate higher ORR and OER catalytic activities (see in Table S7, Supporting Information the evaluation of reported aqueous Zn–air batteries for spinel‐based catalysts). Also, Figure 6d displays the discharge polarization curves and associated power density of Zn–air batteries, demonstrating that the NCMO@rGO electrocatalyst had maximum power density of 125.1 mW cm^−2^ at 0.54 V, surpassing that of NCO@rGO and Pt─C + IrO_2_ electrodes (109.2 and 113 mW cm^−2^ at 0.57 V). Afterward, the durability of long‐term charge‐discharge cycling is one of the most crucial aspects of Zn–air batteries and is key to evaluating the rechargeability of air electrodes. The Zn–air battery charge‐discharge potential curve shows exceptional cycle stability for 590 cycles up to 100 h due to the cathode NCMO@rGO electrocatalyst. The charge‐discharge cycle experiments were run at a constant current density of 5 mA cm^−2^, with discharge lasting 5 minutes and charge lasting an additional 5 minutes, as shown in Figure 6e. In contrast, the Pt─C + IrO_2_ cathode can remain stable for longer than 60 hours. Additionally, at a current density of 5 mA cm^−2^, the NCMO@rGO electrode voltage gap between the charge‐discharge cycles was better (0.93 V) than that of the Pt─C + IrO_2_ cathode (0.89 V), demonstrating superior performance of NCMO@rGO to NCO@rGO electrocatalyst (Figure S21a–e, Supporting Information). This further suggests that NCMO@rGO is an efficient bifunctional catalyst for ORR/OER activities and cyclability. It is worth mentioning that NCO@rGO shows a constant charge voltage without the addition of Mo. The performance of the Zn–air battery is significantly enhanced by surface modification of NCMO@rGO because it contains the most active atoms in the electrocatalyst, as confirmed by in situ XRD analysis. Finally, the entire electrochemical system can be described in terms of the redox processes that occur between the cathode and anode in alkaline solutions (Equations (1), (2), (3)).

(1) $$\begin{array}{ccc} & & \mathrm{Cathode}\;\;\mathrm{reaction}:O_{2}+2H_{2}O+4e^{-}\\ & & \;⇌4OH^{-}\left(E^{o}=0.4\;\;V\;\;\mathrm{versus}\;\;\mathrm{SHE}\right) \end{array}$$

(2) $$\begin{array}{ccc} & & \mathrm{Anode}\;\;\mathrm{reaction}:\mathrm{Zn}+4OH^{-}-2e^{-}\\ & & \;⇌\mathrm{Zn}{\left(\mathrm{OH}\right)}_{4}^{2-}\left(E^{o}=-1.25\;\;V\;\;\mathrm{versus}\;\;\mathrm{SHE}\right) \end{array}$$

(3) $$\mathrm{Overall}\;\;\mathrm{reaction}:\mathrm{Zn}+1/2\;\;O_{2}⇌\mathrm{ZnO}\;\;\left(E^{o}=+\;\;1.65\;\;V\right)$$

### 2.9 In Situ XRD in Zn‐Air Battery

Metal‐air batteries are attractive options for future storage technology because of their desirable theoretical energy density. Alkaline electrolytes, porous carbon cathodes, and zinc metal anodes are commonly used to create aqueous cells, which are frequently used in Zn–air battery systems.^[^ ^64^ ^]^ Herein, the Zn/NCMO@rGO dispersion of the electrode was assessed by in situ synchrotron XRD. The experiments were conducted in transmission mode, and Figure 6f shows the concentrated beam position inside the cell. To cover the diffraction angle 2θ without relocating the detector, a one‐dimensional catalyst‐coated solid beryllium detector was positioned behind the cell. The battery was charged to 3.0 V and discharged to 0.2 V over 65 h in charge‐discharge experiments using a cell cycle at room temperature for constant current density. The typical XRD pattern of our Zn/NCMO@rGO cell assembly exhibits peaks due to the Zn and Be in the counter and working electrodes. In situ XRD patterns of various cathode electrodes were produced before beginning the Zn–air battery discharge (Figure S22a,b, Supporting Information).

The patterns of the NCMO@rGO air cathode electrode before and after stability treatment with 6 M  KOH containing zinc acetate (0.2 M ) electrolyte are compared in Figure S22c (Supporting Information). As can be seen, the ex situ NCMO@rGO disk and NCMO@rGO powder patterns match the experimental results (JCPDS‐016‐0309). Metallic Mo and NCO bands are preserved in the powder NCMO@rGO sample. However, noticeable changes were observed when a Zn‐NCMO@rGO sample was dissolved in a solution of 6 M  KOH containing zinc acetate (0.2 M ). Specifically, new peaks at 27.2°, 38.0°, 39.6°, 44.6°, 46.0°, and 48.7° replaced the NCMO@rGO peaks at 27.9°, 31.7°, 32.7°, and 46.1°. The new JCPDS‐04‐006‐7762 shows that these new peaks can be classified as metallic Mo. Additionally, the production of ZnO (Zn→Zn^2+^)^[^ ^65^ ^]^ was attributed to the peaks at 31.7°, 34.4°, and 47.6° (Figure 6g). Even though our electrodes consist of NCMO@rGO and metallic Zn instead of ZnO, we observed metallic Be peaks in our uncharged state (Figure S22d, Supporting Information). This result indicates that Mo^3+^ must be reduced when Zn is oxidized to Zn^2+^, suggesting a spontaneous redox reaction (Zn↔Zn^2+^, Mo↔Mo^3+^). In addition, Mo^3+^ intermediate products may develop in this reaction, most probably as surface aqueous complexes. Hence, molecules such as Mo(OH)_2_ are produced before Zn oxidation, converting Mo^3+^ to metallic Mo. In contrast, metallic Be peaks develop while NCMO@rGO ones are lost after Zn‐NCMO@rGO electrodes are dissolved in a 6 M  KOH solution containing zinc acetate (0.2 M).

### 2.10 Morphology Characterization for after Zn–air Battery

After in situ XRD stabilization, the morphological changes were studied by using TEM images to identify the reason for the reduced Zn–air battery activity. In contrast to the air‐cathode catalyst, which completely changes the HNF structures, the electrolyte nearly completely changed its original shape (Figure S23a, Supporting Information). Additionally, HR‐TEM images after a charge‐discharge stability study revealed that NCMO@rGO particle crystallinity was well preserved, and the crystal planes (100) and (311) are depicted in Figure S23b (Supporting Information). HAADF‐TEM mapping and an EDX spectrum were used to confirm the HNF NCMO structural changes and associated elements, as depicted in Figure S23c–h (Supporting Information).

To examine changes in surface species of NCMO as activity decreased, the sample was examined using XPS after Zn–air battery charge‐discharge stability tests. As shown, XPS indicated significant changes in the NCMO@rGO in the survey spectrum (Figure S24a, Supporting Information).^[^ ^66^ ^]^ The 2p_3/2_ peaks at the catalyst surface for Ni and Co species were nearly identical, whereas the 2p_1/2_ peaks for NCO and NCMO on rGO electrocatalysts changed significantly (Figure S24b,c, Supporting Information). Interestingly, both of the peaks of 2p_3/2_ were slightly higher after charge–discharge stability, which might improve the Zn–air battery. Mo atoms were more stable than Ni and Co atoms for subsequent stabilization. As shown in Figure S24d (Supporting Information), the 3d_5/2_ and 3d_3/2_ peaks did not shift noticeably in the NCMO@rGO composite of Mo XPS spectrum. Furthermore, as seen in Figure S24e,f (Supporting Information), the O and C spectrum is usually created by the formation of additional hydroxides. Atomic percentages of the NCMO@rGO and NCO@rGO electrocatalysts before and after stability are presented in Figure S25a–d (Supporting Information). Overall, we believe that the density of dopant atoms on oxide catalysts has been associated with the increase in electrochemical activity.^[^ ^67^ ^]^ Future research will focus on finding novel techniques to regulate transition metals and surface defects in catalysts.

## 3 Conclusion

Using a simple hydrothermal process, we effectively developed and produced an HNF structure of NCMO on an rGO base. The obtained Mo atoms exhibited excellent catalytic properties for Zn–air batteries and also excellent electrocatalytic spinel catalysts ability to carry out both bifunctional ORR and OER reactions. The prepared catalysts were studied using operando XAS and in situ XRD analysis. Changes in the catalyst structure and reaction kinetics were investigated by DFT calculations. Also, the ORR and OER reactive processes that occur at metal sites on the NCMO@rGO surface are further discussed in this study. Among, NCMO@rGO show low overpotentials (332 mV) for OER and a half‐wave potential for ORR of just 0.81 V in a 0.1m KOH solution. Moreover, the NCMO@rGO exhibited exceptional ability as a zinc‐air battery and air cathode as well because of its higher OCV, high power density (1.41 V, 125.1 mW cm^−2^), superior specific capacity (976 mAh g^−1^), and long‐term cycle stability (100 h). Finally, the present results open great scope for further improvements to increase catalytic activity and add new features for specific applications such as energy storage.

## 4 Experimental Section

### Materials

Graphite powder (99.99% trace metals basis), nickel (II) nitrate hexahydrate (99.99%), cobalt (II) chloride (98.0%), sodium molybdate dihydrate (99%), and ammonium fluoride (99.99%) were purchased from Sigma Aldrich. Urea (99.0–100.5%), potassium hydroxide (95.0%), zinc acetate (98.0–101.0%), benchmark 20% Pt─C, commercial IrO_2_ (99.9%), and a 5% Nafion solution were obtained from Alfa Aesar. Samchun Pure Chemicals Co. in South Korea provided the methanol, ethanol, and isopropanol.

### Synthesis of Hierarchical Nanoflowers Trimetallic Oxide

Graphite powder was processed using a modified Hummers method to create graphene oxide (GO).^[^ ^68^ ^]^ A suitable concentration of graphene oxide was dissolved in 10 mL of deionized water (10 mg L^−1^), and the mixture was sonicated for 1 h. Before adding the mixture to the GO solution, 20 mL of deionized water (DI) was used to dissolve 1 mmol each of nickel (II) nitrate hexahydrate, cobalt (II) chloride, and sodium molybdate dehydrate and 2 mmol of urea. To the above solution was added 1 mmol diluted ammonium fluoride solution via drop‐wise addition, and then the solution was stirred for 30 min. After that, the mixture was transported to a stainless‐steel autoclave with a Teflon liner that held 40 ml, and the hydrothermal reaction was carried out for 5 hours at 150 °C. To create NiCoMoO_4_ hierarchical nanoflowers structures on rGO, the as‐obtained material was repeatedly washed with DI water and ethanol after being dried at 60 °C overnight, then annealing for 1 h at 200 °C in an air medium. The production of NiCo_2_O_4_@rGO was carried out using the same methodology without Mo precursor.

### Materials Characterization

A high‐resolution transmission electron microscope (HR‐TEM) (JEM‐ARM200F, JEOL) and field emission scanning electron microscope (FE‐SEM) with energy dispersive x‐ray spectroscopy (EDS) (SUPRA 40 VP; Carl Zeiss, Germany) were used to characterize the morphologies of all electrocatalysts produced. The generated electrocatalysts X‐ray diffraction (XRD) patterns were assessed using a PANalytical (X'PERT‐PRO Powder) (model) and Cu K light ( = 0.154 nm). Loading of Ni, Co, and Mo was evaluated using inductively coupled plasma‐optical emission spectrometry (ICP‐OES) on a Thermo Fisher Scientific iCAP 7000 series. The Raman spectrum of each manufactured electrocatalyst was measured using elevated 3D mapping scanning Raman spectroscopy with a NANO PHOTON (RAMAN Touch) outfitted with a 532 nm helium‐neon laser at the Center for University‐wide Research Facilities (CURF) of South Korea Jeonbuk National University (JBNU). The chemical state of the as‐obtained materials was examined using X‐ray photoelectron spectroscopy (XPS; Axis‐Nova, Kratos Inc.). A Brunauer‐Emmett‐Teller (BET) Autosorb‐iQ 2ST/MP physisorption analyzer was used to look at the prepared catalyst surface area, at the South Korean KBSI (Korea Basic Science Institute of Jeonju Center).

### X‐Ray Absorption Spectroscopy Characterization

The synchrotron X‐ray absorption spectroscopy (XAS) analysis were conducted at the Taiwan Light Source (TLS), a part of the National Synchrotron Radiation Research Center (NSRRC), Taiwan, on beamline BL17C, which was fitted with a Si (111) double‐crystal monochromator. The TLS storage ring ran at 1.5 GeV and a current of 360 mA. There were two distinct zones of XAS, and the X‐ray absorption near‐edge structures (XANES) were at the absorption edge of the XAS spectrum between −30 and 100 eV. The oxidation state and band occupancy were determined based on the binding energy of a core electron. The extended X‐ray absorption fine structures (EXAFS), which were oscillations within the region beyond the absorption edge of 50 eV to 1000 eV, were linked to the local electronic structure such as coordination numbers, bond distances, as well as Debye‐Waller factors. These could be produced by electron backscatter by atoms in the surrounding coordination environment. Both the Ni and Co K‐energy edges resolutions were adjusted to 0.35 eV. Transmission mode was used to record the XAS spectra. Standard techniques were used to examine the raw data, including data prior to and after edge background subtractions, edge jump normalization, and Fourier processing.

### In Situ X‐Ray Absorption Spectroscopy Characterization

Operando Ni K and Co K XAS measurements were performed at the National Synchrotron Radiation Research Center (NSRRC) beamline 44A of the Taiwan Photon Source (TPS). The TPS storage ring operated at 3.0 GeV with a current <500 mA. Operando XAS data were collected in transmission mode within desirable conditions with custom‐made operando XAS equipment. The Quick‐XAS mode was used (120 spectra per minute) and 240 unaltered spectra were aggregated to obtain the superior X‐ray absorption fine structure (XAFS) spectra.

## Conflict of Interest

The authors declare no conflict of interest.

## Author Contributions

R.S.K.; Conceptualization, experimental, investigation, formal analysis, and writing‐review original draft. P.M., T.T.T.Nga, and C.‐L.D.; performed ex situ and in situ XAS analysis, data visualization, and wrote the XAS part. J.‐L.Chen; Formal analysis of XAS part. Y.‐S.K.; performed In situ XRD analysis and data visualization. S.P. and D.H.K.; performed the computational simulations and wrote the DFT part. D.J.Y.; supervision of work and review of the original draft, acquisition funding, and research supervision of work.

## Supporting information

Supporting Information

## Data Availability

The data that support the findings of this study are available from the corresponding author upon reasonable request.
