# Supplementary material for: Trimetallic Oxide Electrocatalyst for Enhanced Redox Activity in Zinc–Air Batteries Evaluated by In Situ Analysis
Source: Adv Sci (Weinh). 2023 Oct 2;10(32):2303525. doi: 10.1002/advs.202303525 (PMC10646265; doi:10.1002/advs.202303525)
Supplement: Supplementary file 1 — Supporting Information [file ADVS-10-2303525-s001.pdf]

ADVANCED SCIENCE
Open Access

Supporting Information

for *Adv. Sci.*, DOI 10.1002/advs.202303525

Trimetallic Oxide Electrocatalyst for Enhanced Redox Activity in Zinc–Air Batteries Evaluated by In Situ Analysis

*Ramasamy Santhosh Kumar, Pandian Mannu, Sampath Prabhakaran, Ta Thi Thuy Nga, Yangsoo Kim, Do Hwan Kim, Jeng-Lung Chen, Chung-Li Dong and Dong Jin Yoo**

Supporting Information

Trimetallic Oxide Electrocatalyst for Enhanced Redox Activity in Zinc-Air Batteries Evaluated by In Situ Analysis

Ramasamy Santhosh Kumar[1], Pandian Mannu[2], Sampath Prabhakaran[3], Ta Thi Thuy Nga[2]
Yangsoo Kim[4], Do Hwan Kim[1,5], Dr. Jeng-Lung Chen[6], Chung-Li Dong[2], Dong Jin Yoo[1,7*]

[1]Department of Energy Storage/Conversion Engineering of Graduate School (BK21 FOUR), Hydrogen and Fuel Cell Research Center, Jeonbuk National University, Jeonju, Jeollabuk-do 54896, Republic of Korea

[2]Research Center for X-ray Science, Department of Physics, Tamkang University, Tamsui 25137, Taiwan

[3]Department of Nano Convergence Engineering, Jeonbuk National University, Jeonju, 54896 Jeonbuk, Republic of Korea

[4]Korea Basic Science Institute, Jeonju Center, Jeollabuk-do 54896, Republic of Korea

[5]Division of Science Education and Institute of Fusion Science, Jeonbuk National University, Jeonju-si, Jeollabuk-do 54896, Republic of Korea

[6]National Synchrotron Radiation Research Center, Hsinchu 30076, Taiwan

[7]Department of Life Science, Jeonbuk National University, Jeonju, Jeollabuk-do 54896, Republic of Korea

*Corresponding Email ID: djyoo@jbnu.ac.kr

Fax: +82-(0) 63-270-3909 Tel: +82-(0) 63-270- 3608

Contents:

Figures 1 to 25

Tables 1 to 7

References

2.4. Electrochemical measurements

2.4.1. For ORR measurements. Using rotating ring-disk electrode spinner (RRDE-3A, ALS Co., Japan) attached to the a Gamry Reference 600 electrochemical workstation was used to measure the ORR catalytic activities. The working electrode, reference electrode, and counter electrodes used were RDE (5 mm: 0.19625 cm^2), Ag/AgCl, and graphite rod. Each experiment involved calibrating the reference electrode against RHE. 3 mg of catalyst and 30 μL of 5% Nafion solution were dissolved in 0.5 mL of isopropanol and DI water (1:1) solution, and the mixture was then sonicated for 60 minutes to create a homogenous ink. Afterwards, a rotating disk electrode was drop-coated with 15 μL of catalyst ink (RDE; 5.0 mm in diameter). To compare, we generated catalyst ink using a comparable method and coated it with an RDE electrode. This catalyst ink has a 20-weight percent Pt/C composition and is offered for sale by Johnson-Matthey in the UK. With such a N_2 or O_2 saturated 0.1 M KOH electrolyte, cyclic voltammetry (CV: scan rate 50 mV s^{-1}) as well as linear-sweep voltammetry (LSV: 10 mV s^{-1}) been performed. LSV was also measured using RDE rotating rates among 400 and 2800 rpm using 0.2 and 0.8 V vs. Ag/AgCl. For 30 minutes before to every ORR experiment, the electrolyte being bubbled with oxygen. To maintain O_2 saturation during the measurements, O_2 flow was maintained at a constant rate. A 5000-cycle durability test was performed using the cyclic voltammetry method in a 0.1 M KOH environment with a steady scan rate of 500 mV vs. Ag/AgCl at 1600 rpm.

2.4.2. Calculation for number of electron transfer during ORR. Koutecky–Levich (K–L) plots were used to determine the number of electrons transferred at various potentials. (J^{-1} vs $\omega^{-1/2}$)[1]

$$\frac{1}{J} = \frac{1}{J_L} + \frac{1}{J_K} = \frac{1}{B\omega^{1/2}} + \frac{1}{J_K} \qquad (1)$$

$$B = 0.62\, nF\, C_0\, D_0^{\frac{2}{3}}\, \nu^{-1/6} \qquad (2)$$

Where, J, J_L and J_k are measured current density, diffusion-limiting current density and kinetic –limiting current density, respectively.

F= Faraday constant (F = 96485 C mol^{-1})

ω= Angular velocity for RDE (rad s^{-1})

$\boldsymbol{D_O}$ Oxygen diffusion co-efficient 1.9×10^{-5} cm^2 s^{-1}

$\boldsymbol{C_O}$ =Saturated oxygen concentration of 1.2×10^{-3} mol L^{-1} in 0.1 M KOH

n = number of electron transfer during the ORR

ν kinetic viscosity of the electrolyte (0.01 cm^2 s^{-1})

All the measured potential vs Ag/AgCl ($E_{Ag/AgCl}$) were convert in to potential vs standard RHE (E_{RHE}) by using following universal Nernst equation (3).

$$E_{RHE} = E_{Ag/AgCl} + 0.059pH + E°_{Ag/AgCl} \qquad (3)$$

Where $E°_{Ag/AgCl}$ = 0.1976 at 25 C and pH of 0.1M (ORR measurement) and 0.1M KOH (OER measurement)

2.4.3. For OER measurements. 3 mg of electrocatalyst and 5% Nafion were combined with 30 μL of a 1:1 isopropyl alcohol/DI water solution, sonicated for 60 minutes, and the result was a homogenous ink. The generated ink was applied to a carbon paper surface area of about 1 cm^2, and it was allowed to dry for 12 hours at 60 °C in a drying oven. Furthermore, the active ingredients were injected at a density of around 3 mg cm^{-2}. Similar processes were used to create catalyst inks made of commercialized IrO_2 (99.9%; Sigma-Aldrich) with Pt/C (20 wt%) that have been coated on carbon paper to comparative testing. The OER electrochemical performance was evaluated using a new three-electrode cell design with a working electrode, reference electrode, and counter electrode were using catalyst-coated carbon paper, Ag/AgCl and a graphite rod.

Experimental OER polarization curves were recorded at a constant scanning rate of 1 mV s^{-1}. Using a resonant frequency of 0.01 to 106 Hz and a potential magnitude of 5 mV, electrochemical impedance spectroscopy (EIS) was carried out on the synthesized electrocatalysts. In the non-Faradic region of the CV curve, a double-layer capacitance (C_{dl}) values of the generated catalyst were evaluated to use a scanning rate of 10 to 100 mV s^{-1}. The middle of the potential was defined as the disparity between the densities of the cathodic and anodic currents. The slope of current density with respect to scan rate was twofold as steep as that of C_{dl}.

Calculation of ECSA

$$ECSA = \frac{C_{dl}}{C_s} \quad (4)$$

ECSA = Electrochemical active surface area (ECSA)

C_{dl} = Double layer capacitance

C_s = Specific capacitance (0.040 mF cm^{-2})

2.4.4. Turnover Frequency Calculations (TOF). TOF, which is used to compare the intrinsic activity of several catalysts, is defined as the frequency of the reaction per active site. Typically, the equation: is used to determine the TOF value for OER.

$$\mathrm{TOF} = \frac{\mathrm{j} \times \mathrm{A} \times \eta}{4 \times \mathrm{F} \times \mathrm{n}} \quad (5)$$

where A is the electrode's geometric area, j is the current density following iR compensation, n is the molar number of active sites, η is the Faradic efficiency, F is the Faraday constant. In our work, Mo, Ni, and Co were taken to be the active sites for NCMO@rGO and NCO@rGO catalysts, and the number n was estimated using total loading mass using the equation:

$$n = \frac{m \times NA}{Mw} \quad (6)$$

where m is the loading mass, NA is Avogadros constant, and Mw is the molar mass of the catalysts.

2.4.5. Zinc-air Battery Test. The catalytic inks NCMO@rGO and NCO@rGO were deposited on carbon paper foam, the amount of catalyst was maintained at 3 mg cm^{-2}. An air cathode, 0.25 mm thick (Alfa Aesar, UK) zinc foil, as well as an electrolyte of 6 M KOH and 0.2 M Zn $(CH_3COO)_2$ were used to build a zinc-air battery. Durability testing for extensive charge-discharge cycles were looked into using a Gamry 600 electrochemical workstation. An air cathode electrode constructed of Pt-C (20 wt%) and IrO_2 with a weight ratio of 1:1 was also created using a similar procedure.

Using Pt-C + IrO_2, NCO@rGO, and NCMO@rGO as the air cathode, equations 7 and 8 were used to find the power density (mW cm^{-2}) and specific capacity (mAh g^{-1}) using zinc-air batteries.[2]

Power density (mW cm^{-2}) = Voltage × current density (7)

Specific capacity (mAh g^{-1}) = current × service hours/weight of consumed Zn (8)

2.5. Computational method. The electrocatalyst was further thoroughly evaluated by DFT calculations using the Vienna Ab initio Simulation Package(VASP)[3],[4] based on the information given by XRD results. Grimme's DFT-D3 functional and the Perdew-Burke-Ernzerhof (PBE) exchange-correlation functional were used with a semiempirical GGA type theory.[5] Ion-electron interactions were studied using the projector augmented wave method (PAW), which was employed in VASP.[6] A vacuum spacing of 15 Å was applied for NCMO and NCO to avoid interaction between adjacent atoms. Plane-wave basis set 400 eV was fixed as cut-off energy. The structures were relaxed totally until the Hellman-Feynman forces were lower

than 0.02 eV/Å. For energy and DOS calculations the script "VASPKIT" in the VASP program generates a KPOINT file to calculate the k-point mesh.[7]

Formation energy (E_F) is defined as in eq. 9 below:

$$E_{\mathrm{F}} = E^{*} - \sum_i n_i E_i$$

where E^*, n_i, *and* E_i denote the energy of each crystal structure, number of elements in the crystal, and energy of each constituent element, respectively.

The ORR process can be decomposed into the following elementary steps:

$$^{*} + O_2 + H^{+} + e^{-} \rightarrow OOH^{*} \quad , \quad (9)$$

$$OOH^{*} + H^{+} + e^{-} \rightarrow O^{*} + H_2O \quad , \quad (10)$$

$$O^{*} + H^{+} + e^{-} \rightarrow OH^{*} \quad , \quad (11)$$

$$OH^{*} + H^{+} + e^{-} \rightarrow H_2O \quad , \quad (12)$$

where * represents the active site on the surfaces of the catalysts.

For each elementary step of ORR, the free energy has been calculated according to the method proposed by Nørskov et al. The free energy change from initial state to final state of the reaction is defined as: $\Delta G = \Delta E + \Delta E_{ZPE} - T\Delta S + \Delta G_U + \Delta G_{pH}$, where ΔE represents the reaction energy difference of reactant and product, which can be directly calculated from DFT computations. ΔE_{ZPE} and ΔS are the changes in zero-point energies and entropy at room temperature (T = 298.15 K), which can be computed from the vibrational frequencies. ΔG_{pH} is the correction on the pH in the electrolyte, which can be determined by $\Delta G_{pH} = k_B T \ln 10 \times pH$. Based on previous theoretical studies, [8],[9],[10] the value of pH in this work was assumed to be zero for acidic

medium. The Gibbs free energy of O_2 ($G_{O2)}$ will be obtained by $G_{O2} = G_{H2O} - 2G_{H2} + 4.92$ eV, because the DFT method fails to accurately describe the high spin ground state of the O_2 molecule. The free energy change for the four elementary ORR steps can be obtained as:

$$\Delta G_1 = \Delta G_{OOH*} \quad , \quad (13)$$

$$\Delta G_2 = \Delta G_{O*} - \Delta G_{OOH*} \quad , \quad (14)$$

$$\Delta G_3 = \Delta G_{OH*} - \Delta G_{O*} \quad , \quad (15)$$

$$\Delta G_4 = 4.92 - \Delta G_{OH*} \quad , \quad (16)$$

Therefore, the overpotential (η) that evaluates the performance of OER, and ORR is applied according to the following equations:

$$\eta_{ORR} = \max\{\Delta G_1, \Delta G_2, \Delta G_3, \Delta G_4\}/e^{-} + 1.23 \quad , (17)$$

where 1.23 represents the equilibrium potential. Based on previous reports, a lower η value on a given catalyst suggests a less energy input for ORR, thus demonstrating its higher ORR catalytic activity.

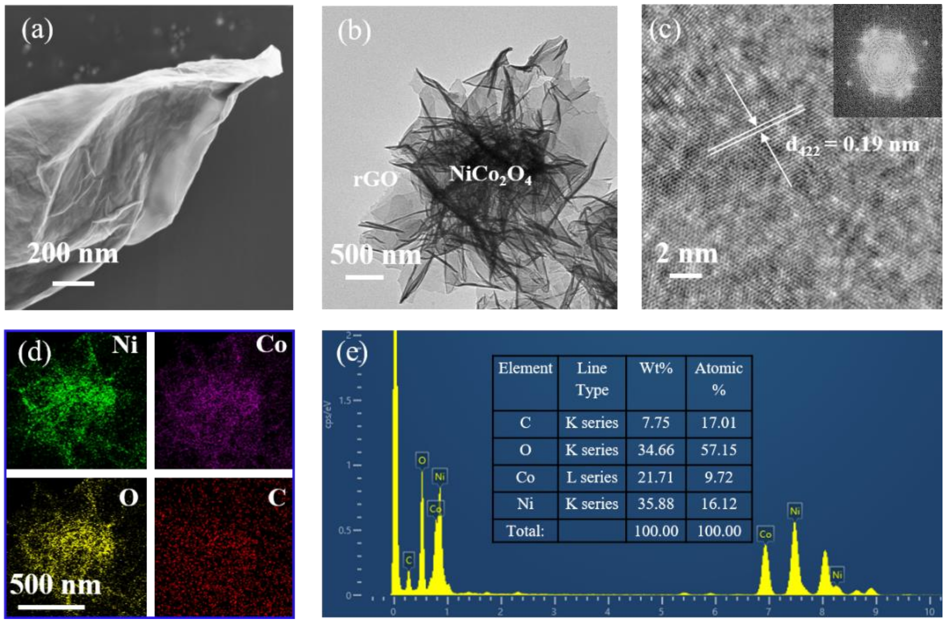

Figure S1. SEM image of (a) GO, (b, c) TEM and HR-TEM images of NCO@rGO. (d, e) TEM elemental color mapping and TEM-EDS spectrum of NCO@rGO catalyst.

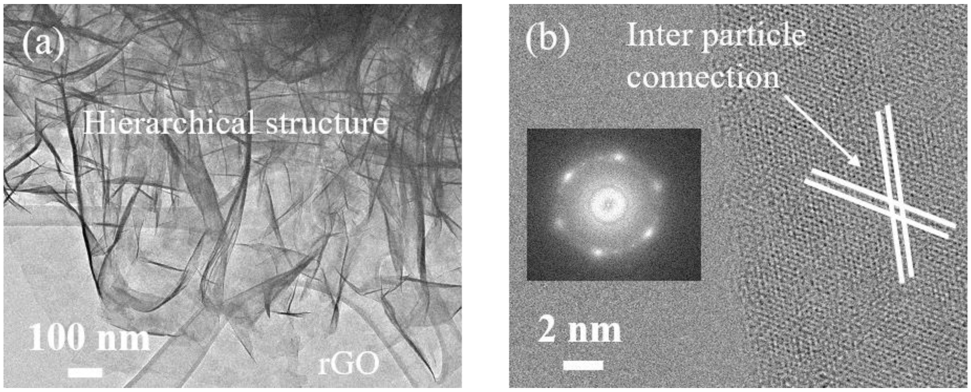

Figure S2. (a, b) TEM and HR-TEM (inset image: FFT pattern) images of NCMO@rGO catalyst.

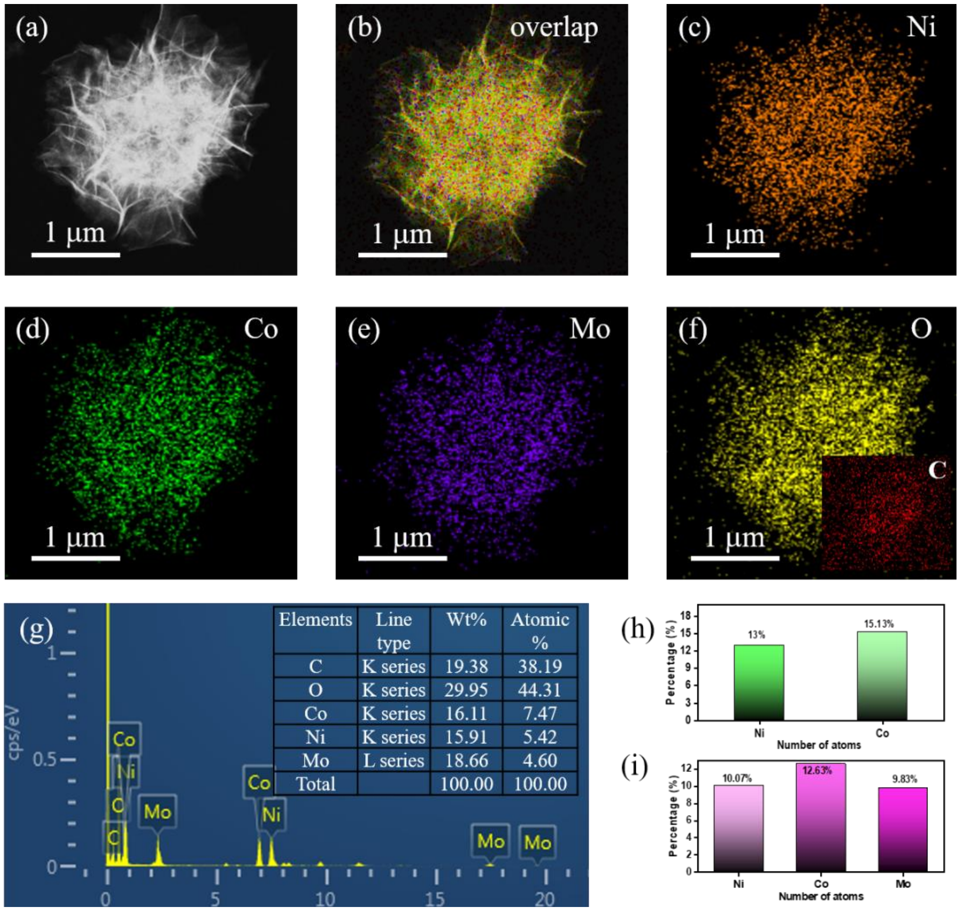

Elements	Line type	Wt%	Atomic %
C	K series	19.38	38.19
O	K series	29.95	44.31
Co	K series	16.11	7.47
Ni	K series	15.91	5.42
Mo	L series	18.66	4.60
Total		100.00	100.00

Figure S3. (a-f) TEM color mapping, (g) TEM-EDX spectrum of NCMO@rGO catalyst. (h, i) ICP-OEC analysis of NCO@rGO and NCMO@rGO catalysts, respectively.

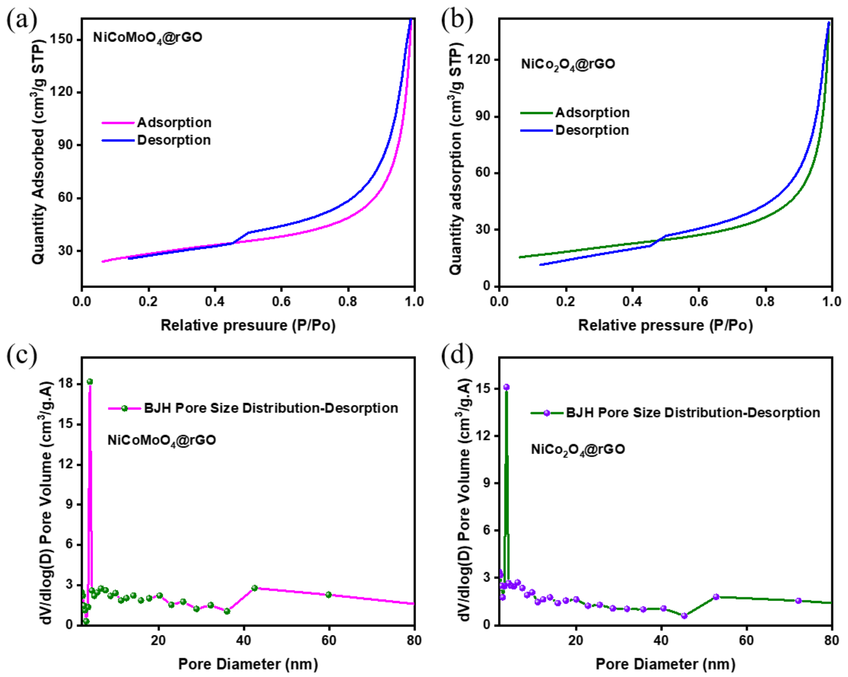

Figure S4. (a, b) BET surface area measured from N_2 adsorption-desorption isotherms and (c, d) pore volume distribution from desorption isotherms of NCMO@rGO and NCO@rGO catalysts.

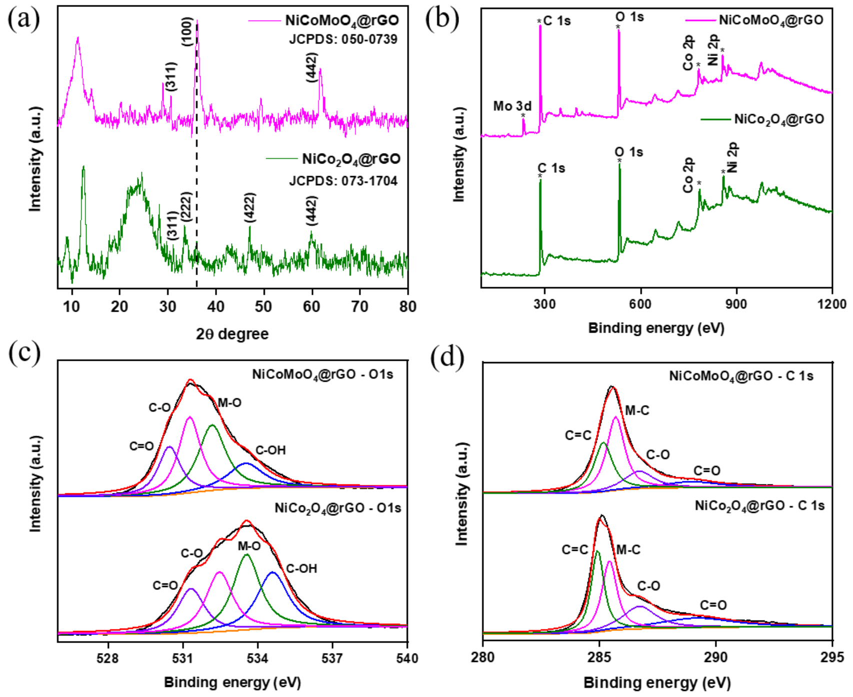

Figure S5. (a) X-ray diffraction analysis of NCO@rGO, and NCMO@rGO catalysts. (b) XPS survey spectra, (c, d) O 1s and C 1s spectra of NCO@rGO, NCMO@rGO catalysts, respectively.

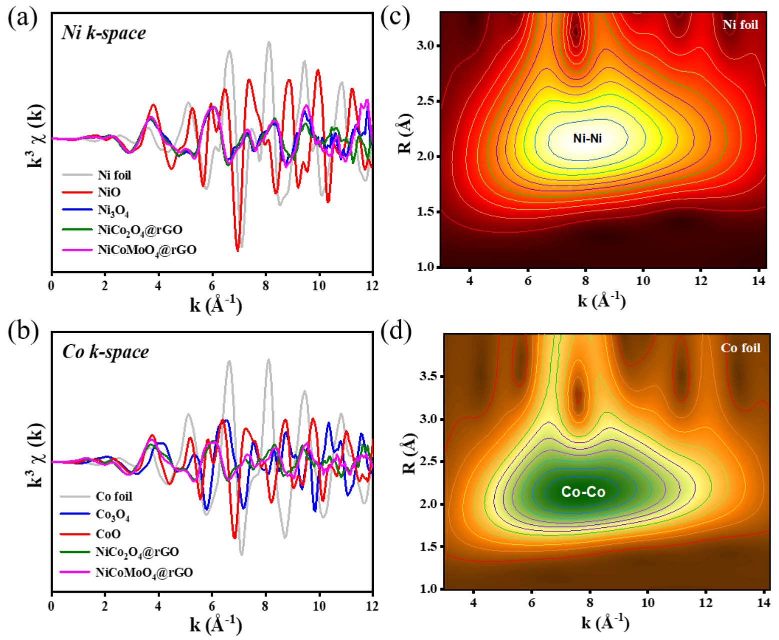

Figure S6. (a-b) EXAFS of Co and Ni k-space for NCO@rGO, NCMO@rGO catalysts with comparison catalysts. (c-d) WT transform of Ni and Co foil.

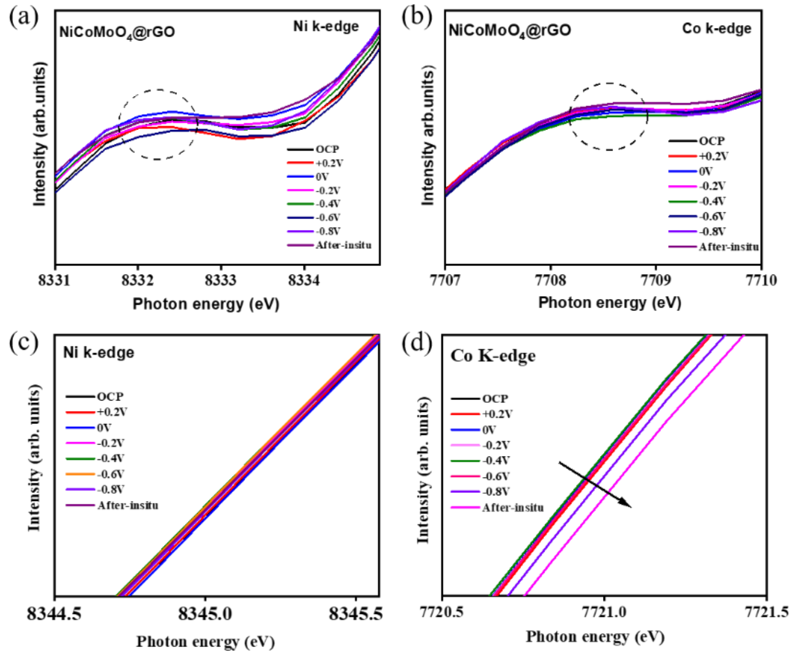

Figure S7. In-situ XANES spectra pre-edge peak at Ni K-edge (a) and (b) Co K-edge of $NiCoMoO_4$@rGO electrocatalyst with different potential. In-situ XANES spectra rising-edge peak at Ni K-edge (c) and (d) Co K-edge of $NiCoMoO_4$@rGO electrocatalyst with different potential.

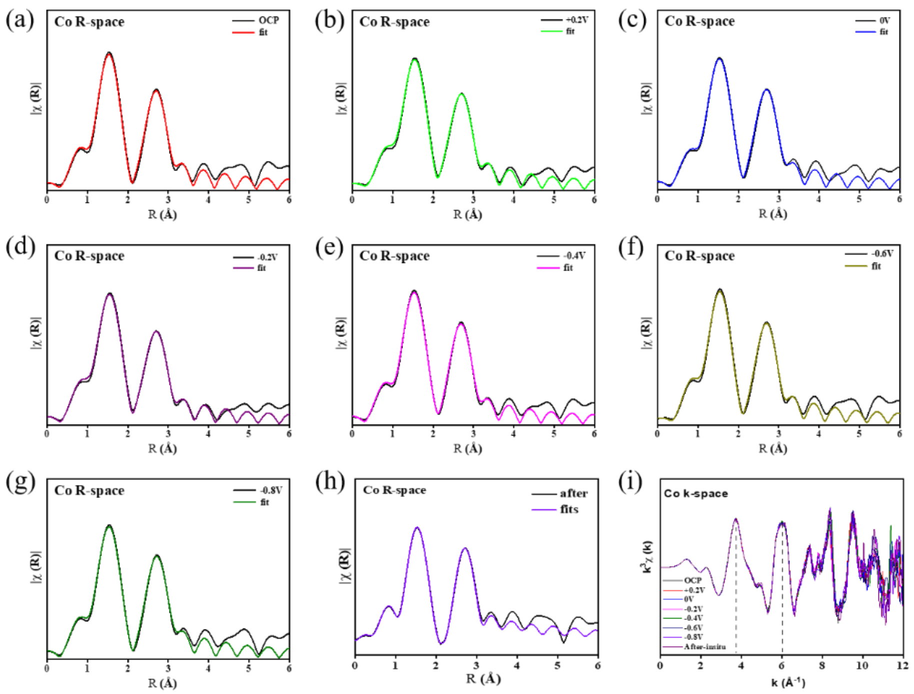

Figure S8. (a-h) The fitting results of the Co K-edge EXAFS spectra at OCP and -0.80 V applied potential. (i) In-situ EXAFS spectra of Co K-edge NCMO@rGO electrocatalyst.

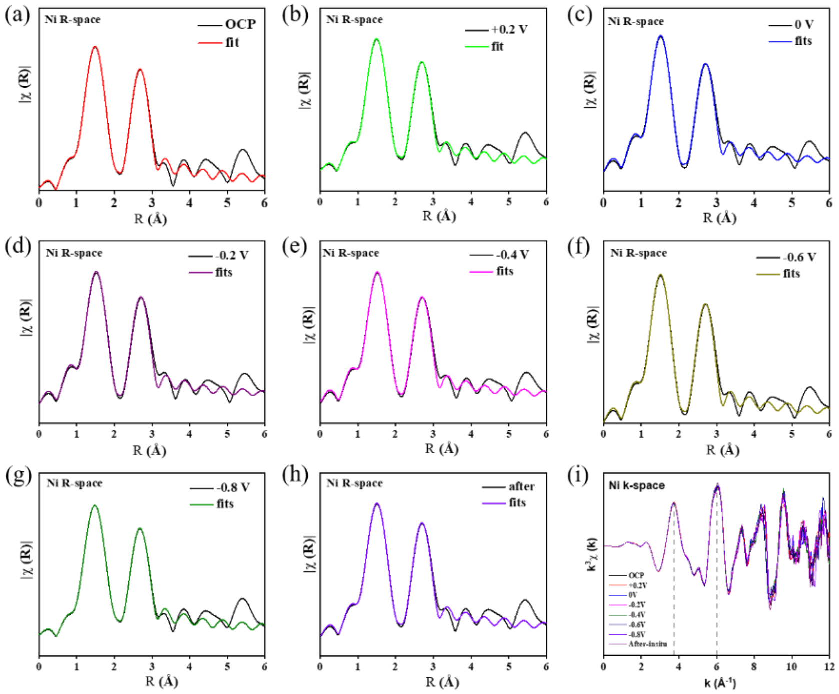

Figure S9. (a-h) The fitting results of the Ni K-edge EXAFS spectra at OCP and -0.80 V applied potential. (i) In-situ EXAFS spectra of Ni K-edge NCMO@rGO electrocatalyst.

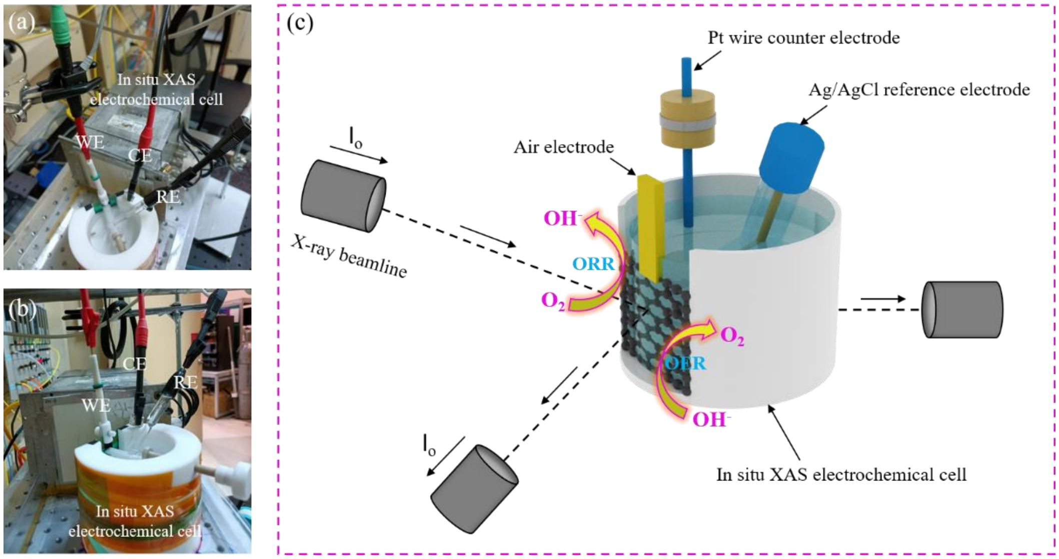

Figure S10. (a-c) Typical experimental set-up for an in situ XAS experiment on an ORR reaction. The experiment has been conducted at Taiwan Photon Source (TPS) beamline 44A of the National Synchrotron Radiation Research Center (NSRRC).

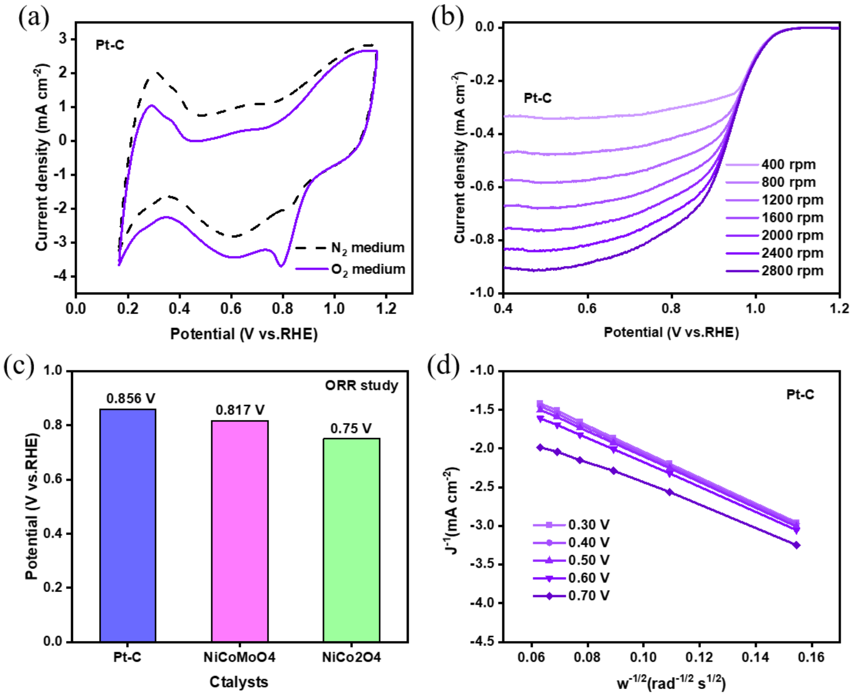

Figure S11. (a) CV curves, (b) LSV curves, (c) comparison of ORR study of prepared catalyst, (d) Koutecky-Levich plots of benchmark Pt-C electrocatalyst.

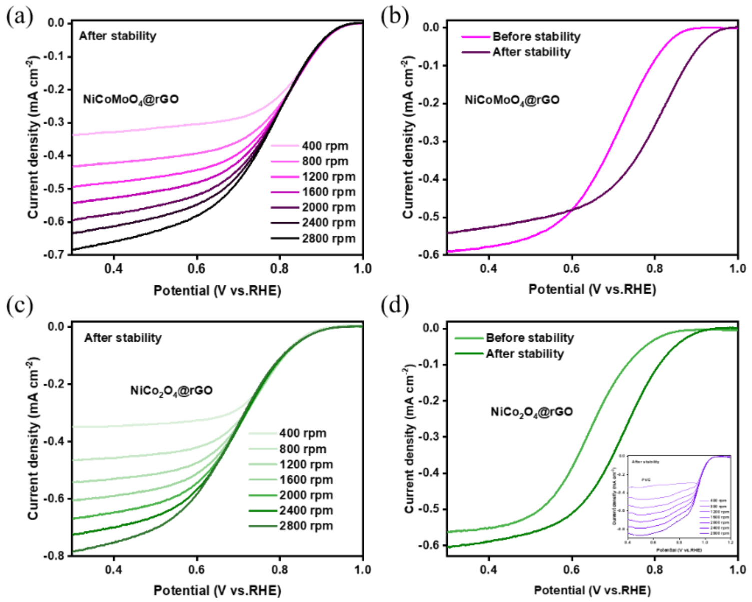

Figure S12. For ORR study: (a, c) after stability LSV curves for 400-2800 rpm speed, (b, d) before and after stability LSV curves for 1600 rpm speed of NCMO@rGO and NCO@rGO electrocatalysts (inset image: Pt/C after stability different rpm speed LSV curves).

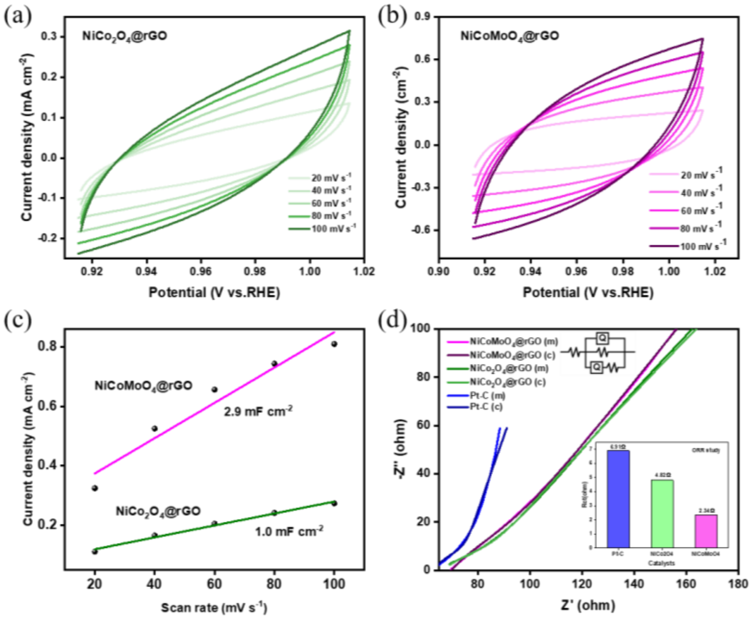

Figure S13. For ORR study: (a, b) CV curves with different scan rates, (c) double-layer capacitance NCO@rGO, and NCMO@rGO. (d) Nyquist plots of Pt-C, NCO@rGO, and NCMO@rGO electrocatalysts (inset image: charge transfer resistance).

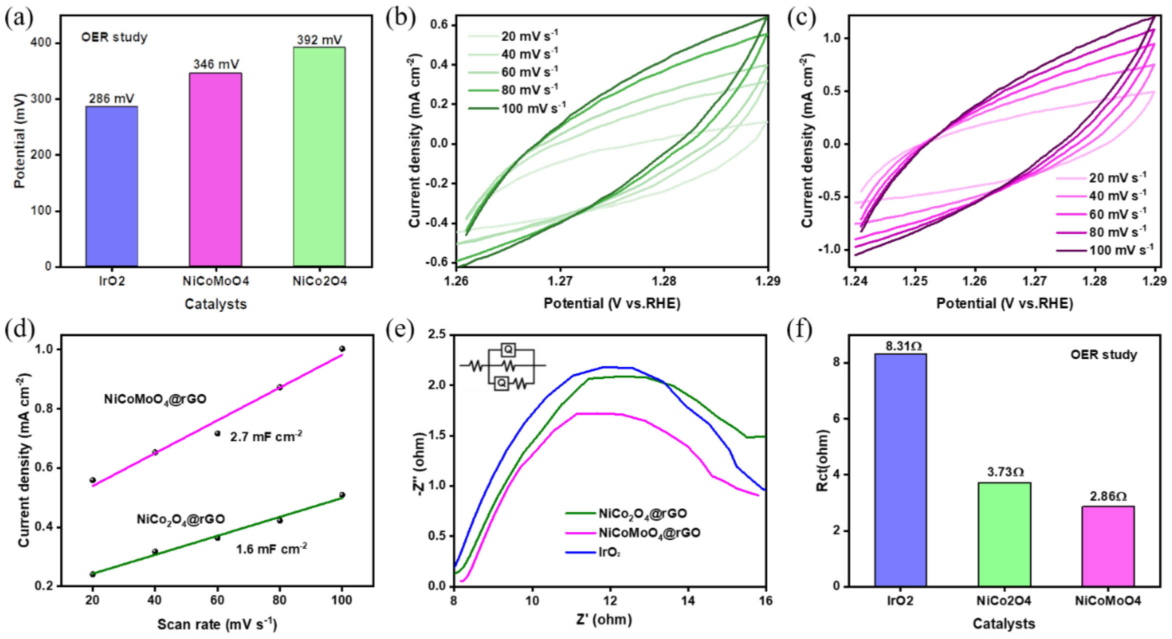

Figure S14. For OER study: (a) comparison table, (b, c) cyclic voltammetry curves with different scan rates, (d) double-layer capacitance, (e) Nyquist plots, and (f) charge transfer resistance of IrO_2, NCO@rGO, and NCMO@rGO electrocatalysts, respectively.

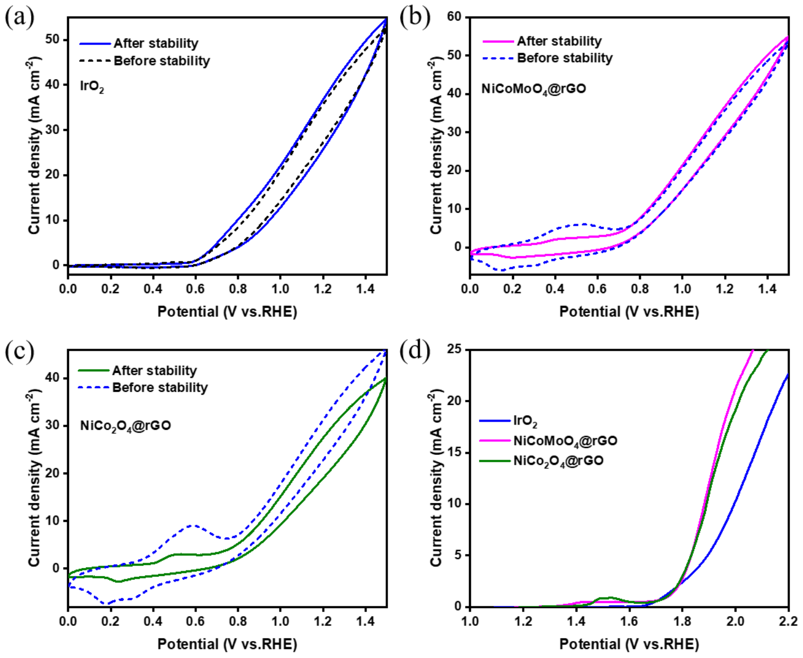

Figure S15. For OER study: (a-c) before and after stability CV curves, (d) After cyclic stability LSV curves of IrO_2, NCO@rGO, and NCMO@rGO electrocatalysts, respectively.

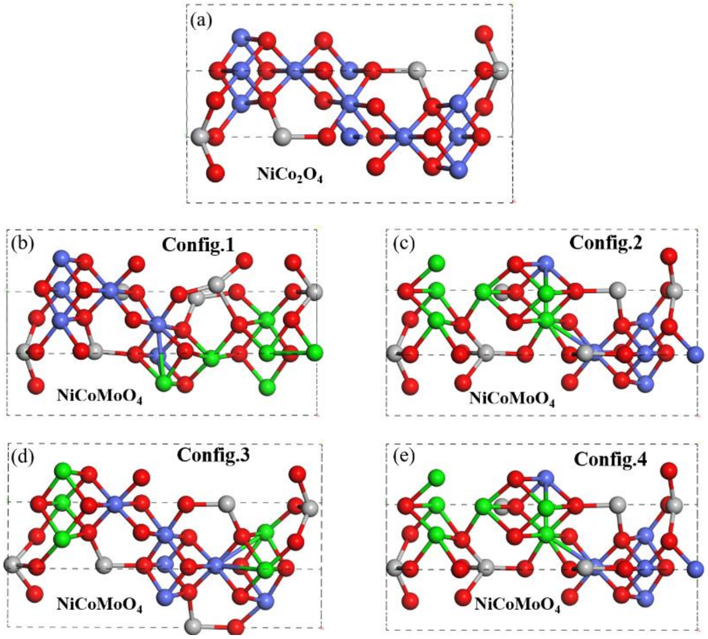

Figure S16. (a) Bulk structure of NCO and, (b-e) NCMO at different configurations of the catalyst. The relative formation energy (Ef, eV) is given in Table(S1) with respect to the most stable structure (config.2).

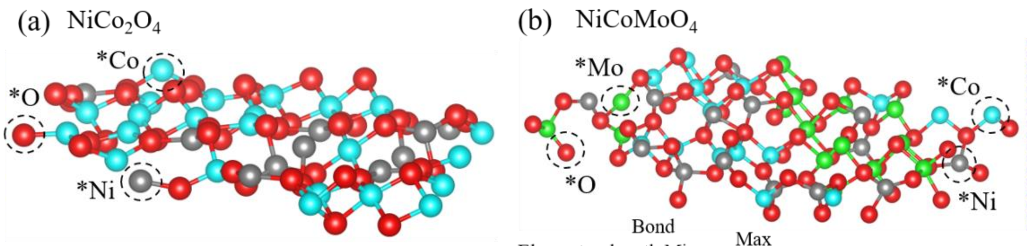

Element	Bond length Min (Å)	Max (Å)
Ni-O	1.714	2.080
Co-O	1.592	2.112
Co-Ni	2.464	2.728

Element	Bond length Min (Å)	Max (Å)
Mo-O	1.521	2.113
Ni-O	1.641	1.915
Co-O	1.561	1.944
Mo-Ni	2.753	2.818
Co-Ni	2.943	3.070
Mo-Co	2.588	2.981

Figure S17. The optimized bulk structure, the catalyst was cleaved at the 311 planes with Expected bond length of (a) NCO and (b) NCMO catalysts, respectively.

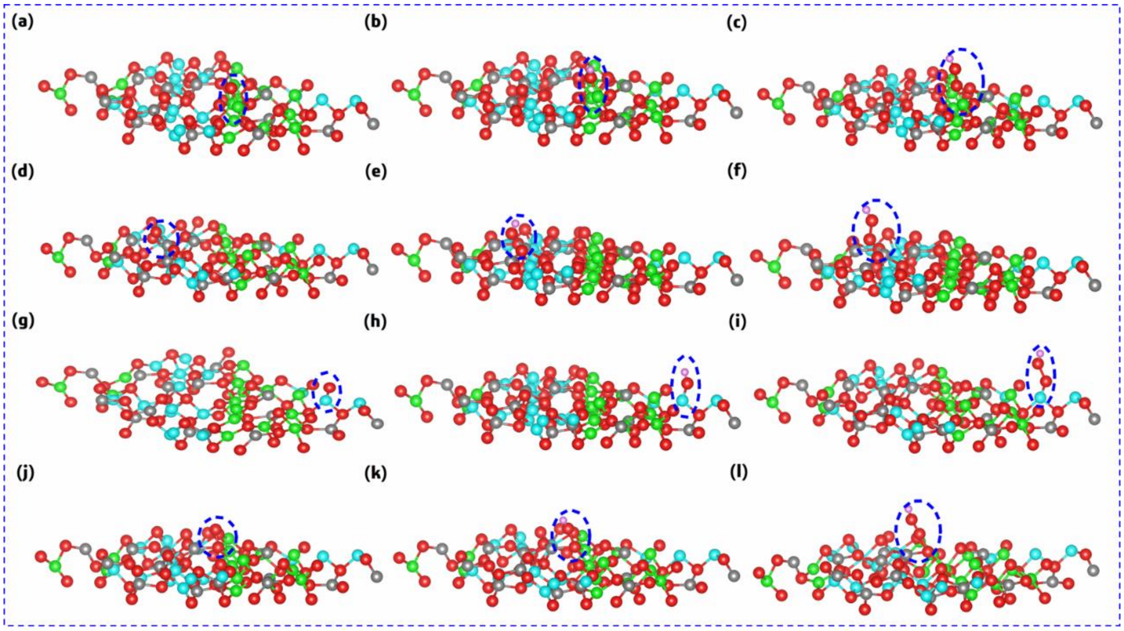

Figure S18. Adsorption structures of the OER surface intermediates (O*, HO*, HOO*) for the NCMO at 311 planes. The adsorption sites are (a-c) *Mo, (d-f) *Ni, (g-i) *Co and, (j-l) *O. Green, Grey, Cyan, Red and Pink colors represent Mo, Ni, Co, O and H atoms, respectively.

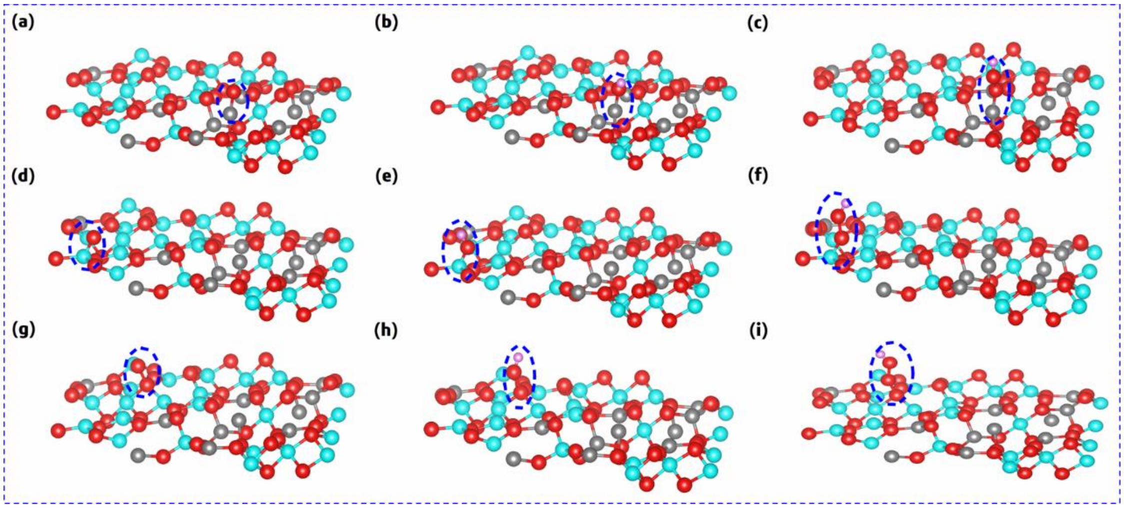

Figure S19. Adsorption structures of the OER surface intermediates (O*, HO*, HOO*) for the NCO at 311 planes. The adsorption sites are (a-c) *Ni, (d-f) *Co and, (g-i) *O. Green, Grey, Cyan, Red and Pink colors represent Mo, Ni, Co, O and H atoms, respectively.

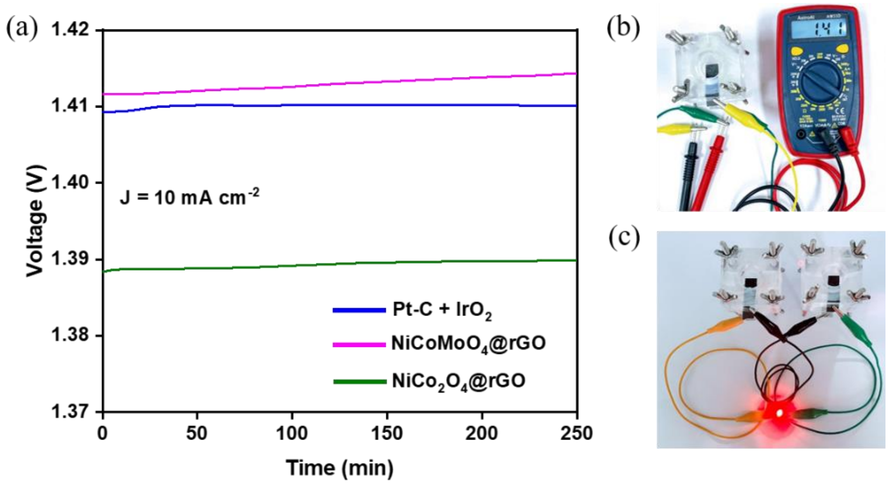

Figure S20. Zn-air battery performance: (a) OCV curve, (b) image of battery with OCV, (c) image of LED light in using Zn-air battery.

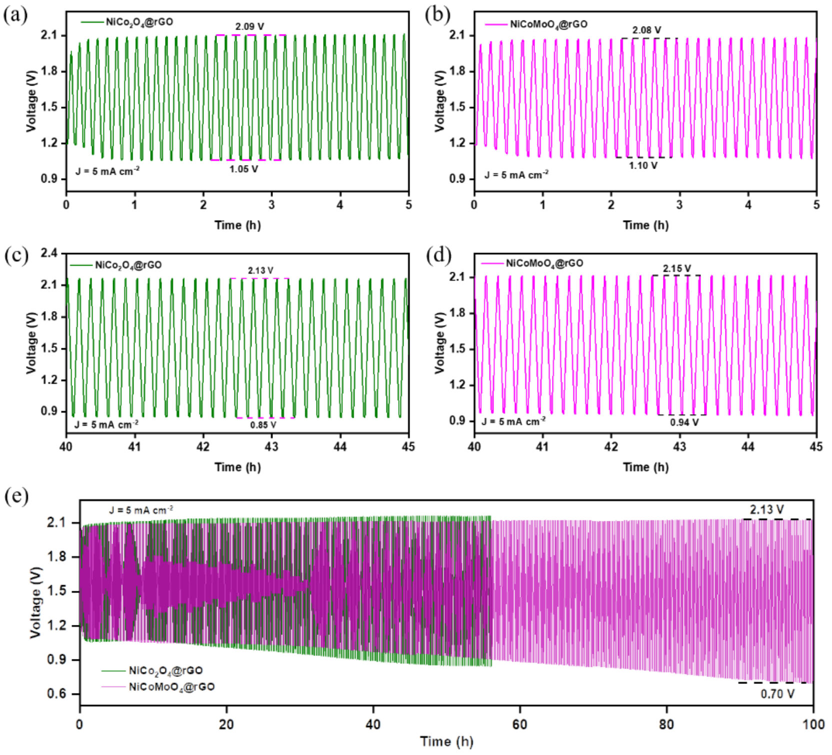

Figure S21. (a-d) Potential difference galvanostatic charge-discharge curves, (e) long-term galvanostatic charge-discharge curves of the Zn-air batteries based on the NCO@rGO and NCMO@rGO air cathodes at current density 5 mA cm^{-2}.

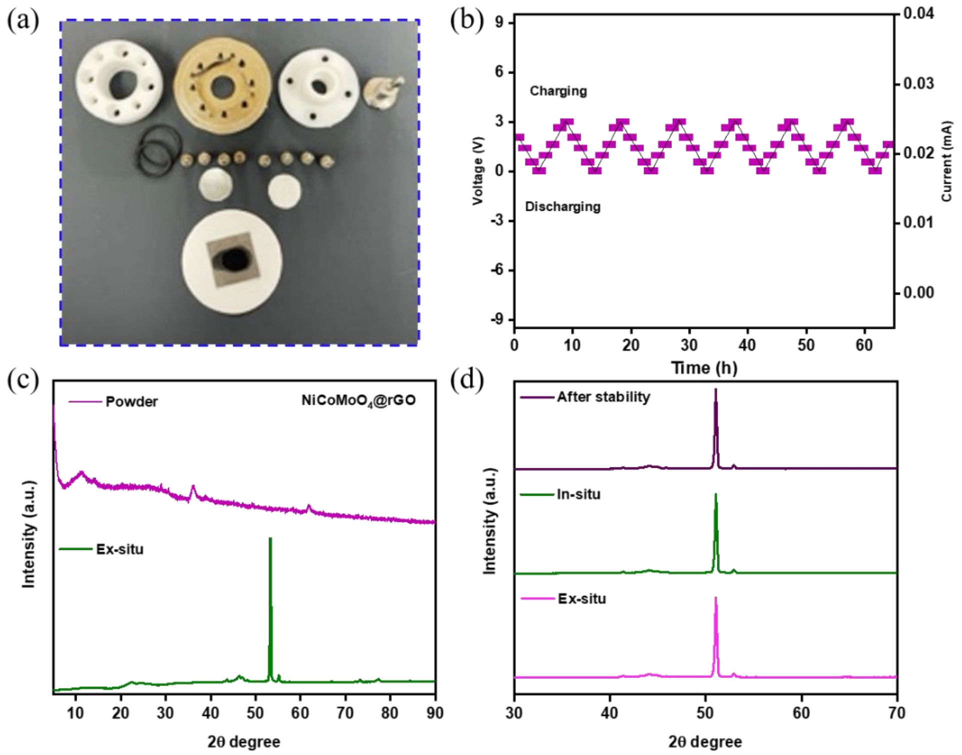

Figure S22. (a) Divided Test Cell for In-Situ XRD Analysis of Battery Electrode with Beryllium (Be) sheet, (b) charge-discharge curves of the Zn-air batteries at current density 10 mA cm^{-2}, (c) conventional XRD patterns, (d) comparison of XRD pattern of NCMO@rGO electrocatalyst.

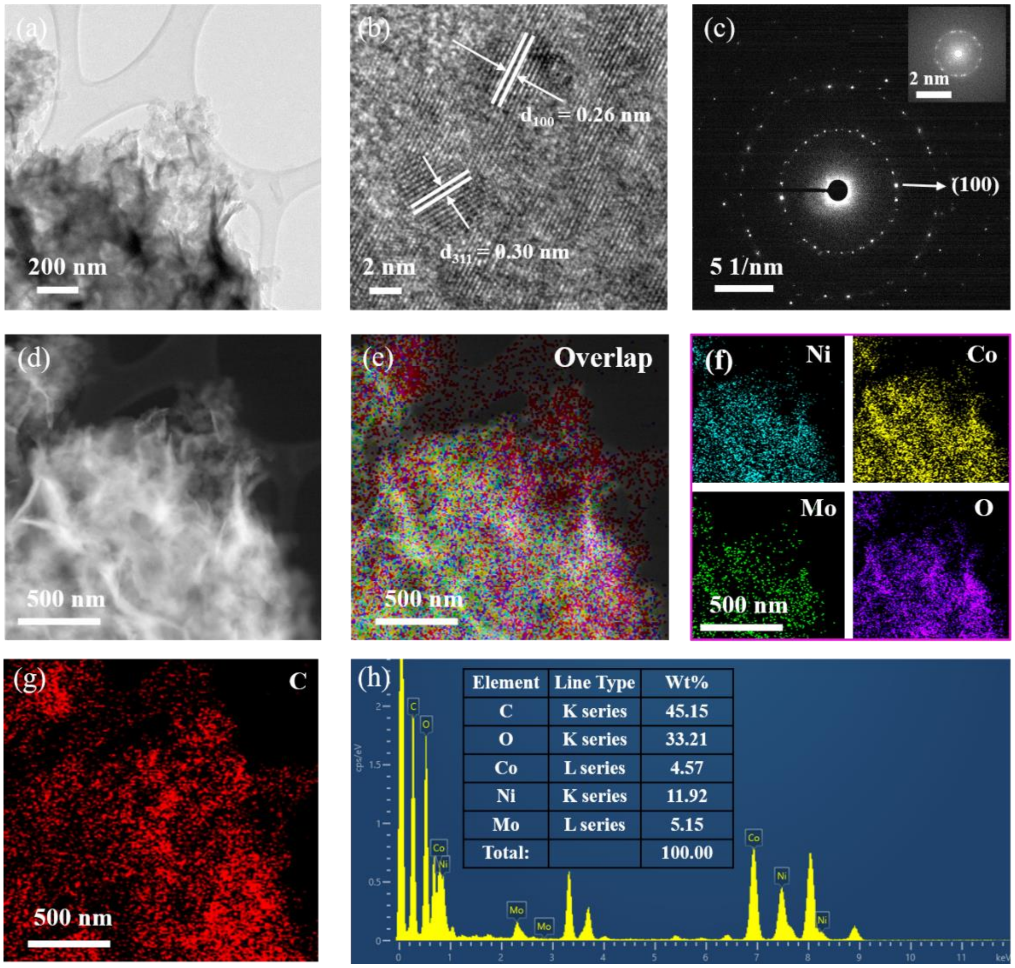

Figure S23. (a) TEM, (b) HR-TEM, and (c) SAED images of $NiCoMoO_4$@rGO catalyst for after Zn-air battery charge-discharge stability (inset image; FFT pattern), (d-g) HAADF-TEM image and corresponding elemental color mapping, (h) EDX spectrum of $NiCoMoO_4$@rGO corresponding elemental percentage.

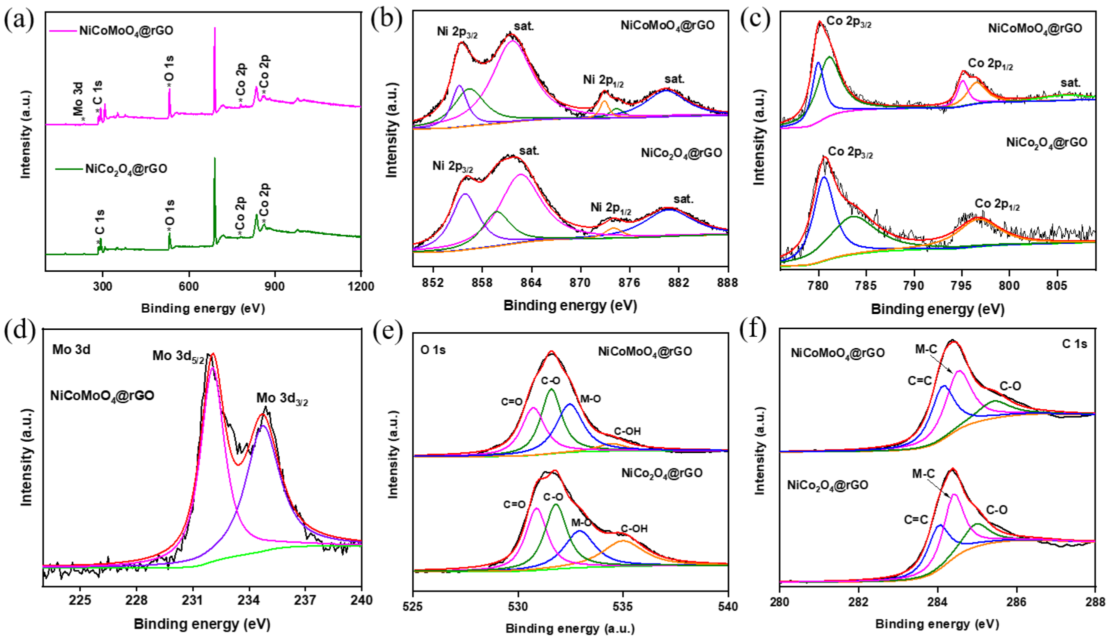

Figure S24. After Zn-air battery stability XPS analysis; (a) survey spectrum, (b) Ni 2p, (c) Co 2p, $NiCo_2O_4$@rGO, $NiCoMoO_4$@rGO catalysts, (d) Mo 3d spectra of $NiCoMoO_4$@rGO catalyst, (e) O1s, and (f) C 1s spectrum $NiCo_2O_4$@rGO, $NiCoMoO_4$@rGO catalysts, respectively.

(a)

Name (before)	Peak BE	Atomic %
C1s	285.13	61.12
O1s	533.37	30
Co 2p	783.39	4.23
Ni 2p	857.94	4.65

(c)

Name (before)	Peak BE	Atomic %
Mo3d	232.36	1.25
C1s	285.52	65.05
O1s	531.44	26.53
Co 2p	781.39	3.03
Ni 2p	856.08	4.14

(b)

Name (after)	Peak BE	Atomic %
C1s	284.32	41.25
O1s	531.55	55.34
Co2p	780.7	1.8
Ni $2p_{3/2}$	855.06	1.04
Ni $2p_{1/2}$	872.84	0.57

(d)

Name (after)	Peak BE	Atomic %
Mo3d	231.99	0.87
O1s	531.59	91.91
Co2p	780.32	3.91
Ni $2p_{3/2}$	855.04	1.94
Ni $2p_{1/2}$	872.8	1.38

Figure S25. Before and after Zn-air battery charge-discharge stability, XPS peak tables of (a, b) $NiCo_2O_4$@rGO and (c, d) $NiCoMoO_4$@rGO catalysts, respectively.

Table S1. The details of the fitting of the EXAFS spectra of Co K-edge, showing the bond distance.

	Bond	$NiCoMoO_4$ @rGO	CN= N × S_0^2	σ^2 ($Å^{-2}$)	ΔE_0 (eV)	R-factor	R(Å)
**OCP**	Co-O	Path_1_[O]	5.32	0.01093	-7.834	0.0055	2.03
	Co-Co	Path_2_[Co]	5.92	0.01499	-6.926		3.09
**+0.20 V**	Co-O	Path_1_[O]	5.27	0.01116	-7.163	0.0035	2.04
	Co-Co	Path_3_[Co]	5.89	0.01480	-7.446		3.09
**0 V**	Co-O	Path_1_[O]	5.08	0.01058	-7.036	0.0040	2.04
	Co-Co	Path_3_[Co]	5.92	0.01431	-6.967		3.09
**-0.20 V**	Co-O	Path_1_[O]	5.34	0.01132	-7.411	0.0050	2.04
	Co-Co	Path_3_[Co]	5.91	0.01454	-7.909		3.09
**-0.40 V**	Co-O	Path_1_[O]	5.19	0.01121	-6.361	0.0027	2.04
	Co-Co	Path_3_[Co]	5.87	0.01521	-7.261		3.09
**-0.60 V**	Co-O	Path_1_[O]	5.09	0.01084	-6.527	0.0041	2.04
	Co-Co	Path_3_[Co]	5.89	0.01451	-7.538		3.09
**-0.80 V**	Co-O	Path_1_[O]	5.02	0.01040	-7.26	0.0045	2.03
	Co-Co	Path_3_[Co]	5.90	0.01423	-6.05		3.11
**After**	Co-O	Path_1_[O]	5.00	0.00941	-8.575	0.0064	2.02
	Co-Co	Path_3_[Co]	5.89	0.01339	-6.711		3.09

Table S2. The details of the fitting of the EXAFS spectra of Ni K-edge, showing the bond distance.

	Bond	$NiCoMoO_4$ @rGO	CN= N × S_0^2	σ^2 ($Å^{-2}$)	ΔE_0 (eV)	R-factor	R(Å)
**OCP**	Ni-O	Path_1_[O]	5.33	0.00564	-9.756	0.0032	2.01
	Ni-Ni	Path_2_[Co]	5.96	0.00954	-8.250		3.07
**+0.20 V**	Ni-O	Path_1_[O]	5.32	0.00539	-9.646	0.0032	2.02
	Ni-Ni	Path_3_[Co]	5.96	0.00946	-7.717		3.07
**0 V**	Ni-O	Path_1_[O]	5.32	0.00520	-9.840	0.0020	2.01
	Ni-Ni	Path_3_[Co]	5.95	0.00988	-7.245		3.08
**-0.20 V**	Ni-O	Path_1_[O]	5.31	0.00562	-9.077	0.0022	2.02
	Ni-Ni	Path_3_[Co]	5.95	0.00991	-7.529		3.08
**-0.40 V**	Ni-O	Path_1_[O]	5.32	0.00550	-9.292	0.0021	2.02
	Ni-Ni	Path_3_[Co]	5.94	0.00979	-7.663		3.07
**-0.60 V**	Ni-O	Path_1_[O]	5.33	0.00543	-9.381	0.0025	2.02
	Ni-Ni	Path_3_[Co]	5.93	0.00988	-7.397		3.08
**-0.80 V**	Ni-O	Path_1_[O]	5.33	0.00530	-10.183	0.0069	2.02
	Ni-Ni	Path_3_[Co]	5.92	0.00957	-8.407		3.07
**After**	Ni-O	Path_1_[O]	5.30	0.00535	-9.630	0.0032	2.02
	Ni-Ni	Path_3_[Co]	5.93	0.00917	-7.635		3.07

N is the coordination number; S_0^2 is amplitude reduction factor, R is the interatomic distance (the bond length between X-ray absorbing atoms and surrounding coordination atoms); σ^2 is

Debye-Waller factor (a measure of thermal and static disorder in absorber-scatterer distances); E_0, inner potential correction; *R*-factor, indicating the goodness of the fit.

Table S3 Calculated formation energies of $NiCo_2O_4$ and different configuration of $NiCoMoO_4$.

**Catalyst**	**Formation Energy (E_F), eV.**
$NiCo_2O_4$	-5.339
Config.1 ($NiCoMoO_4$)	-5.467
Config.2 ($NiCoMoO_4$)	-5.488
Config.3 ($NiCoMoO_4$)	-5.486
Config.4 ($NiCoMoO_4$)	-5.468

Table S4 Calculated formation energies of $NiCo_2O_4$ and $NiCoMoO_4$ at 311 planes.

**Catalyst**	**Formation Energy (E_F), eV.**
$NiCo_2O_4$	-4.975
$NiCoMoO_4$	-5.006

Table S5 The calculated Gibbs free energy (ΔG) of the catalysts

**Catalyst**	Adsorption site	ΔG, (eV)				OER Overpotential (η, V)	ORR Overpotential (η, V)
		ΔG_1	ΔG_2	ΔG_3	ΔG_4		
**$NiCo_2O_4$**	Ni	1.455	0.905	2.075	0.485	0.85	0.74
	Co	2.267	1.147	1.102	0.403	1.04	0.82
	O	1.418	0.920	2.591	-0.008	1.36	1.23
**$NiCoMoO_4$**	Mo	0.760	1.813	1.570	0.776	0.58	0.47
	Ni	1.233	1.698	1.424	0.564	0.47	0.66
	Co	1.086	2.350	0.311	1.173	1.12	0.91
	O	0.992	1.907	0.198	1.823	0.68	1.03

Table S6. Summary of the electrochemical performance of various bifunctional spinel catalysts as reported by other researchers. $E_{1/2}$ is half-wave potential for ORR and $E_{i=10}$ is the potential for OER at current density of 10 mA cm^{-2}. The $\triangle E$ ($\triangle E = E_{i=10} - E_{1/2}$) serves as a metric to evaluate bifunctional Electrocatalytic properties. These electrochemical performances are measured in 0.1 M KOH solution.

Catalysts	ORR $E_{1/2}$ (V)	OER $E_{i=10\ mA\ cm^{-2}}$	ΔE ($E_{i=10}$-$E_{1/2}$) (V)	Ref.
**$NiCoMoO_4$@rGO**	**0.81**	**1.562**	**0.75**	**This work**
$Mn_{0.5}Ni_{0.5}Co_2O_4$	0.76	1.63	0.87	[11]
$MnCo_2O_4$@C	0.80	1.66	0.89	[12]
Co_3O_4/CNF	0.85	1.64	0.79	[13]
$NiCo_2O_4$@N-CNWs	0.80	1.56	0.77	[14]
$Co(OH)_2$-$NiCo_2O_4$	0.80	1.63	0.83	[15]
$NiCo_2O_4$–CN	0.81	1.613	0.80	[16]
$NiCo_2O_4$-rGO	0.78	1.611	0.83	[17]
Co_3O_4@$NiCo_2O_4$	0.81	1.65	0.84	[18]
NiO/$NiCo_2O_4$	0.73	1.587	0.85	[19]
$MnCo_2O_4$/CNF	0.83	1.63	0.79	[20]
CoNiMn@500*	0.65	1.72	1.07	[21]
Co@Co_3O_4/NC-2	0.74	1.64	0.90	[22]
$NiCo_2O_4$	0.72	1.75	1.03	[23]
CoO@Co_3O_4/NSG	0.79	1.69	0.90	[24]
PCO/Co_3O_4 NCs	0.72	1.60	0.88	[25]

Table S7. The performance comparison of reported aqueous Zn-Air batteries for spinel-based catalysts.

Catalysts	Metal loading (mg cm^{-2})	OCV (V)	Power density (mW cm^{-2})	Specific capacity (mAh g^{-1})	Cycling stability (h)	Ref.
**$NiCoMoO_4$@rGO**	**3.0**	**1.41**	**125.1**	**976.8**	**100**	**This work**
$Mn_{0.5}Ni_{0.5}Co_2O_4$	2.0	1.38	117	1587	21	[11]
$NiCo_2O_4$@N-CNWs	2.5	1.45	93.02	826.9	212	[14]
$NiCo_2O_4$/CNTs	2.0	1.39	185	769	320	[26]
$NiCo_2O_4$ PNSs	2.0	1.33	83	520	120	[27]
$NiCo_2O_4$/MXene	1.0	1.40	277	768.6	333	[28]
$NiCo_2O_4$/N-G	1.4	-	108.3	792.6	54	[29]
$NiCo_2O_4$/Mo_2C/CC	2.0	1.393	104	778	300	[30]
$MnCo_2O_4$/CNF	1.0	1.4	40	-	10	[20]
CoNiMn@500*	2.0	1.36	49	876.4	10	[21]
PCO/Co_3O_4 NCs	2.0	1.404	182	878	180	[25]
Ce@Co_3O_4/CNF	1.0	1.40	97.7	-	50	[31]
Co-NiO NFs	2.0	1.38	93	830	110	[32]
$CuCo_2O_4$/N-CNTs	2.0	1.36	83.3	817.4	24	[33]
$NiCo_2O_4$/CNTs	2.0	1.36	172	551	10	[34]
$NiCo_2O_4$@NiMn LDH	1.0	1.4	160.8	722	83	[35]

Reference

[1] Y. Liang, Y. Li, H. Wang, J. Zhou, J. Wang, T. Regier, H. Dai, *Nat. Mater.* **2011**, *10*, 780-786.

[2] Z. Qian, Y. Chen, Z. Tang, Z. Liu, X. Wang, Y. Tian, W. Gao, *Nano-Micro Lett.* **2019**, *11*, 28.

[3] A. S. Botana, M. R. Norman, *Physical Review Materials* **2019**, *3*, 044001.

[4] G. Kresse, J. Hafner, *Journal of Physics: Condensed Matter* **1994**, *6*, 8245.

[5] S. Ehrlich, J. Moellmann, W. Reckien, T. Bredow, S. Grimme, *ChemPhysChem* **2011**, *12*, 3414-3420.

[6] P. E. Blöchl, O. Jepsen, O. K. Andersen, *Physical Review B* **1994**, *49*, 16223-16233.

[7] V. Wang, N. Xu, J.-C. Liu, G. Tang, W.-T. Geng, *Computer Physics Communications* **2021**, *267*, 108033.

[8] Y. Qin, M. Yang, C. Deng, W. Shen, R. He, M. Li, *Nanoscale* **2021**, *13*, 5800-5808.

[9] Y. Li, R. Hu, Z. Chen, X. Wan, J.-X. Shang, F.-H. Wang, J. Shui, *Nano Research* **2021**, *14*, 611-619.

[10] H. Zeng, X. Liu, F. Chen, Z. Chen, X. Fan, W. Lau, *ACS Applied Materials & Interfaces* **2020**, *12*, 52549-52559.

[11] J. Béjar, L. Álvarez–Contreras, F. Espinosa–Magaña, J. Ledesma–García, N. Arjona, L. G. Arriaga, *Electrochimica Acta* **2021**, *391*, 138900.

[12] C. Shenghai, S. Liping, K. Fanhao, H. Lihua, Z. Hui, *Journal of Power Sources* **2019**, *430*, 25-31.

[13] C. Alegre, C. Busacca, A. Di Blasi, O. Di Blasi, A. S. Aricò, V. Antonucci, E. Modica, V. Baglio, *Journal of Energy Storage* **2019**, *23*, 269-277.

[14] H. Ge, G. Li, T. Zheng, F. Wang, M. Shao, H. Liu, X. Meng, *Electrochimica Acta* **2019**, *319*, 1-9.

[15] X. Peng, J. Zhang, T. Cen, Z. Ye, Y. Liu, D. Yuan, *Journal of Alloys and Compounds* **2021**, *872*, 159441.

[16] Y. Li, Z. Zhou, G. Cheng, S. Han, J. Zhou, J. Yuan, M. Sun, L. Yu, *Electrochimica Acta* **2020**, *341*, 135997.

[17] Y. Li, G. Cheng, Z. Zhou, X. Liao, S. Han, F. Ye, M. Sun, L. Yu, *ChemElectroChem* **2019**, *6*, 4429-4436.

[18] N.-F. Yu, W. Huang, K.-L. Bao, H. Chen, K. Hu, Y. Zhang, Q.-H. Huang, Y. Zhu, Y.-P. Wu, *Dalton Transactions* **2021**, *50*, 2093-2101.

[19] Z. Zhang, X. Liang, J. Li, J. Qian, Y. Liu, S. Yang, Y. Wang, D. Gao, D. Xue, *ACS Applied Materials & Interfaces* **2020**, *12*, 21661-21669.

[20] C. Alegre, C. Busacca, A. Di Blasi, C. Cannilla, O. Barbera, V. Antonucci, M. J. Lázaro, V. Baglio, *Journal of Energy Storage* **2022**, *55*, 105404.

[21] S. Kosasang, H. Gatemala, N. Ma, P. Chomkhuntod, M. Sawangphruk, *Batteries & Supercaps* **2020**, *3*, 631-637.

[22] A. Aijaz, J. Masa, C. Rösler, W. Xia, P. Weide, A. J. R. Botz, R. A. Fischer, W. Schuhmann, M. Muhler, *Angewandte Chemie International Edition* **2016**, *55*, 4087-4091.

[23] C. Jin, F. Lu, X. Cao, Z. Yang, R. Yang, *Journal of Materials Chemistry A* **2013**, *1*, 12170-12177.

[24] X. Huang, J. Wang, H. Bao, X. Zhang, Y. Huang, *ACS Applied Materials & Interfaces* **2018**, *10*, 7180-7190.

[25] B. He, Y. Deng, H. Wang, R. Wang, J. Jin, Y. Gong, L. Zhao, *Journal of Colloid and Interface Science* **2022**, *625*, 502-511.

[26] X. Xiao, X. Li, J. Wang, G. Yan, Z. Wang, H. Guo, Y. Liu, *Ceramics International* **2020**, *46*, 6262-6269.

[27] J. Yin, J. Jin, H. Liu, B. Huang, M. Lu, J. Li, H. Liu, H. Zhang, Y. Peng, P. Xi, C.-H. Yan, *Advanced Materials* **2020**, *32*, 2001651.

[28] H. Lei, S. Tan, L. Ma, Y. Liu, Y. Liang, M. S. Javed, Z. Wang, Z. Zhu, W. Mai, *ACS Applied Materials & Interfaces* **2020**, *12*, 44639-44647.

[29] Y. Ma, W. Shang, W. Yu, X. Chen, W. Xia, C. Wang, P. Tan, *Energy & Fuels* **2021**, *35*, 14188-14196.

[30] C. Xu, Q. Wang, S. Zhao, S. Wang, *Synthetic Metals* **2021**, *280*, 116894.

[31] X. Sun, T. Xu, W. Sun, J. Bai, C. Li, *Journal of Alloys and Compounds* **2022**, *898*, 162778.

[32] J. Qian, X. Guo, T. Wang, P. Liu, H. Zhang, D. Gao, *Applied Catalysis B: Environmental* **2019**, *250*, 71-77.

[33] H. Cheng, M.-L. Li, C.-Y. Su, N. Li, Z.-Q. Liu, *Advanced Functional Materials* **2017**, *27*, 1701833.

[34] N. Xu, Y. Cai, L. Peng, J. Qiao, Y.-D. Wang, W. M. Chirdon, X.-D. Zhou, *Nanoscale* **2018**, *10*, 13626-13637.

[35] X. Guo, T. Zheng, G. Ji, N. Hu, C. Xu, Y. Zhang, *Journal of Materials Chemistry A* **2018**, *6*, 10243-10252.
